# Hybrid-state free precession in nuclear magnetic resonance

**DOI:** 10.1038/s42005-019-0174-0

**Published:** 2019-06-25

**Authors:** Jakob Assländer, Dmitry S. Novikov, Riccardo Lattanzi, Daniel K. Sodickson, Martijn A. Cloos

**Affiliations:** 1 Department of Radiology, Center for Biomedical Imaging, New York University School of Medicine, New York, NY, USA.; 2 Center for Advanced Imaging Innovation and Research, New York University School of Medicine, New York, NY, USA.; 3 The Sackler Institute of Graduate Biomedical Sciences, New York University School of Medicine, New York, NY, USA.

## Abstract

The dynamics of large spin-1/2 ensembles are commonly described by the Bloch equation, which is characterized by the magnetization’s non-linear response to the driving magnetic field. Consequently, most magnetic field variations result in non-intuitive spin dynamics, which are sensitive to small calibration errors. Although simplistic field variations result in robust spin dynamics, they do not explore the richness of the system’s phase space. Here, we identify adiabaticity conditions that span a large experiment design space with tractable dynamics. All dynamics are trapped in a one-dimensional subspace, namely in the magnetization’s absolute value, which is in a transient state, while its direction adiabatically follows the steady state. In this hybrid state, the polar angle is the effective drive of the spin dynamics. As an example, we optimize this drive for robust and efficient quantification of spin relaxation times and utilize it for magnetic resonance imaging of the human brain.

For many nuclei, the spin gives rise to a magnetic moment, whose dynamics can be exploited, among other things, for quantum computing^1^, to study the chemical structure of molecules, as done in nuclear magnetic resonance^2^ (NMR) spectroscopy, or to analyze the composition of biological tissue, as used for clinical diagnosis in magnetic resonance imaging^3^ (MRI). Modeling spin–lattice and spin–spin interactions as random magnetic field fluctuations^4^ allows for capturing their macroscopic effect by the relaxation times *T*_1_ and *T*_2_, respectively. This facilitates the description of large spin-1/2 ensembles with the classical Bloch equation^5^, formally akin to the time-dependent Schrödinger equation in a 4D-space:

(1) $$\partial_{t}\left(\begin{array}{l} x\\ y\\ z\\ 1 \end{array}\right)=\left(\begin{array}{cccc} -\frac{1}{T_{2}} & -\omega_{z} & \omega_{y} & 0\\ \omega_{z} & -\frac{1}{T_{2}} & -\omega_{x} & 0\\ -\omega_{y} & \omega_{x} & -\frac{1}{T_{1}} & \frac{1}{T_{1}}\\ 0 & 0 & 0 & 0 \end{array}\right)\left(\begin{array}{l} x\\ y\\ z\\ 1 \end{array}\right).$$

Here, ∂_*t*_ denotes the partial derivative with respect to time, *x*, *y*, *z* are the spatial components of the magnetization, and 1 is the normalized *z*-magnetization at thermal equilibrium. The Rabi frequencies^2^ *ω*_*x*_ and *ω*_*y*_ (induced by radio frequency (RF) pulses), together with the Larmor frequency *ω*_*z*_, are the external drive of the spin dynamics.

While the Bloch equation is very general, it provides little intuition to help design robust and efficient experiments. This lack of intuition has biased experimental design towards elementary drives for which analytic solutions make the effect of spin relaxation and experimental imperfections evident. For example, the workhorses of clinical MRI weight the signal intensity either by *T*_1_ or *T*_2_ effects by exploiting the simplest spin dynamics, most notably exponential relaxation^6–8^ and steady states^9–11^. These basic drives span small subspaces like the steady-state ellipse^9,12–14^, which entail relatively trivial spin dynamics compared to the richness found outside. More recent approaches strive to break away from such traditional experimental designs in search for an improved signal-to-noise efficiency^15^. However, the non-intuitive nature of the Bloch equation has limited the exploration of this vast experiment design space to heuristic guesses^15–20^.

The rationale for this improved encoding efficiency can be understood intuitively: Variations of the driving fields result in a transient state, which enables one to exploit the entire Bloch sphere in search for the optimal encoding of characteristic parameters such as spin relaxation times. However, there is a risk associated with the transient state: Small magnetic field deviations can produce substantial differences in spin trajectories, which can bias the estimation of characteristic parameters. This is particularly problematic in biological tissues, where inhomogeneous broadening and diffusion narrowing are inevitable and are nontrivial to model^19,21,22^.

Here, we formulate conditions under which the sensitivity to magnetic field deviations and inhomogeneous broadening is greatly mitigated and reveal a large subspace of drives in which the Bloch equation is tractable. Our analysis shows that, under these conditions, the direction of the magnetization adiabatically follows the one of steady states, while the absolute value of the magnetization can be in a transient state. In this hybrid state, the spin dynamics thus live in a one-dimensional subspace and can be described by a 2 × 2 Hamiltonian:

(2) $$\partial_{t}\left(\begin{array}{l} r\\ 1 \end{array}\right)=\left(\begin{array}{cc} -\frac{{\text{cos}\;}^{2}\;\vartheta }{T_{1}}-\frac{{\text{sin}}^{2}\vartheta }{T_{2}} & \frac{\text{cos}\;\vartheta }{T_{1}}\\ 0 & 0 \end{array}\right)\left(\begin{array}{l} r\\ 1 \end{array}\right),$$

where *r* is the magnetization along the radial direction, i.e. its magnitude (refer to the section “Methods” for the derivation). This notation identifies the polar angle *ϑ*(t), which is the angle between the *z*-axis and the magnetization, as the relevant degree of freedom, which describes the joint effect of the drives *ω*_*x*_(*t*), *ω*_*y*_(*t*), and *ω*_*z*_(*t*) on the spin dynamics. As an example, we show that this hybrid-state equation and its solution provide intuition for the encoding processes of spin-relaxation times and are an excellent basis for numerical optimizations of a *T*_1_ and *T*_2_ mapping experiment that combines the robustness of the steady state with the encoding efficiency of the transient state.

## Hybrid state boundary conditions

**Fig. 1:**
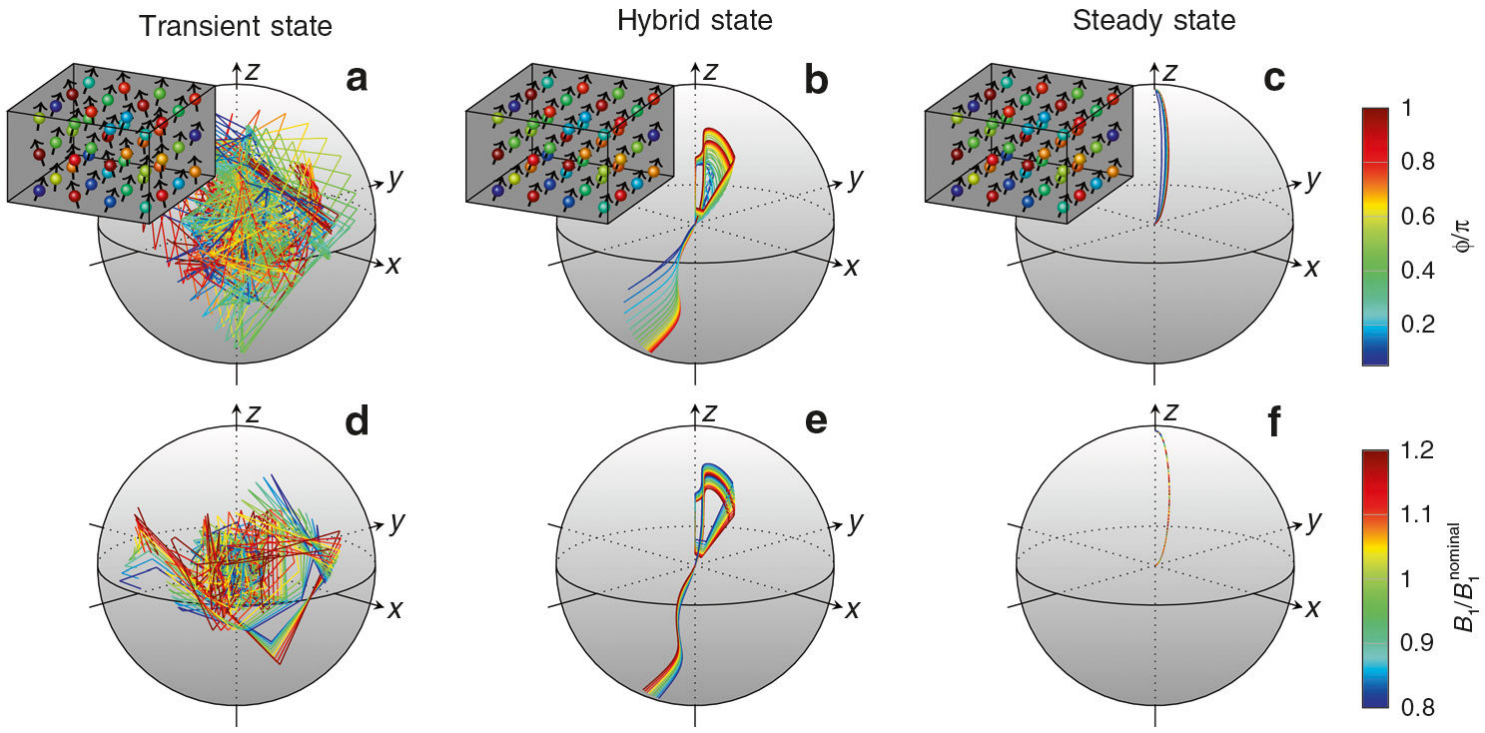
Comparison of spin ensemble states. **a** In a fully transient state (here visualized at the example of a random pattern of radio frequency pulses), the spin trajectories on the Bloch sphere are, in general, very sensitive to magnetic field inhomogeneities. **b** The hybrid state is explicitly designed to mitigate these sensitivities, while still allowing the magnetization to visit the entire Bloch sphere. **c** Fully adiabatic transitions between steady states have the same robustness to magnetic field deviations, however, they trap the magnetization on the steady-state ellipse^9, 12–14^, which diminishes the capability to encode tissue properties such as relaxation times. The steady-state ellipse is described by setting the left-hand side of (Eq. 2) to zero. **a**–**c** Deviations of *B*_0_, which dictates the Larmor frequency, has strong practical implications, because local magnetic field variations in a sample in nuclear magnetic resonance (or in a volume element in magnetic resonance imaging (MRI), also known as a voxel and here visualized as a cube) give rise to a distribution of different Larmor frequencies. Consequently, the observed signal, given by the integral over all measured frequencies, depends on the unknown distribution of Larmor frequencies^23^. Here, *ϕ* denotes the phase accumulated over one repetition time *T*_R_, and we define *ϕ* = *π* as the on-resonance condition. **d**–**f** Deviations of the radio-frequency field *B*_1_, which dictates the Rabi frequencies, can lead to strong variations of the spin trajectories in the transient state. Note that in clinical MRI, the Rabi frequency within a voxel is approximately constant

An eigendecomposition of the Bloch–Hamiltonian points out the source of the sensitivity to magnetic field inhomogeneities. As the magnetization described by (Eq. 1) is real-valued, we can conclude that the eigenvalues of the Hamiltonian must either be real-valued or occur in complex conjugate pairs. One eigenvalue is zero and describes the steady-state magnetization. Therefore, another eigenvalue must be real-valued. As such, it describes an exponential decay of the corresponding transient-state component, while the remaining complex eigenvalues describe oscillatory decays. Ganter pointed out that the complex phase makes the latter components very sensitive to deviations in the magnetic field and, in particular, to inhomogeneous broadening^23^. Figure 1 provides some intuition for this sensitivity: As the complex phase accumulates during the experiment, the spin trajectory becomes very sensitive to deviations in the magnetic fields. Considering that the measured signal is invariably given by the integral over some distribution of Larmor frequencies^21^, or more generally, over the Brownian paths in a magnetically heterogeneous environment^22^, contributions of the complex eigenvalues will lead to a bias in the estimated relaxation parameters^19^.

Conversely, if we design our MR experiment such that the cumbersome complex-valued eigenstates are not populated, we achieve robustness to magnetic field deviations and inhomogeneous broadening. If we simultaneously populate the real-valued transient eigenstate, we liberate the magnetization from the steady-state ellipse and gain access to the entire Bloch sphere (Fig. 1).

In general, variations of the driving fields rotate the eigenvectors and populate all transient eigenstates. A Taylor expansion of this eigenbasis rotation (see the section “Methods”) reveals that this population is dominated by the gaps between the eigenvalue and the rest of the Hamiltonian’s spectrum, similar to the quantum mechanical adiabatic theorem^24^. If we assume *T*_1_ = *T*_2_ as an illustrative example, the Hamiltonian has the eigenvalues *λ*_1_ = 0, *λ*_2_ = −1/*T*_1,2_, and *λ*_3,4_ = −1/*T*_1,2_ ± *i****|ω***| with ***ω*** = (*ω*_*x*_, *ω*_*y*_, *ω*_*z*_). This illustrates that the separation of the real-valued eigenvalue from the steady-state relies on relaxation, while the spin-ensemble’s frequency adds to the separation of the complex-conjugate eigenvalues. It is this structure of the Hamiltonian’s spectrum that enables the hybrid state.

For pulsed experiments^6^, which dominate modern MR, a thorough derivation (see the section “Methods”) results in the steady-state adiabatic condition

(3) $$\text{max}\{|\Delta \alpha |,|\Delta \phi |\}\ll {\left(T_{\text{R}}/T_{1}\right)}^{2}.$$

Here, the driving fields are parameterized by the flip angle *α* and the accumulated phase *ϕ* = *ω*_*z*_*T*_R_, and Δ*α* and Δ*ϕ* denote the change of these parameters in consecutive repetitions. For biological tissue, rapid imaging protocols usually use *T*_R_ ≪ *T*_1_, which makes (Eq. 3) a very restrictive bound.

The eigenvalues’ phase allows for a substantially less restrictive bound for the complex eigenstates:

(4) $$\text{max}\left\{\left|\text{Δ}\alpha |,|\text{Δ}\phi \right|\right\}\ll {\text{sin}}^{2}\frac{\alpha }{2}+{\text{sin}}^{2}\frac{\phi }{2}.$$

**Fig. 2:**
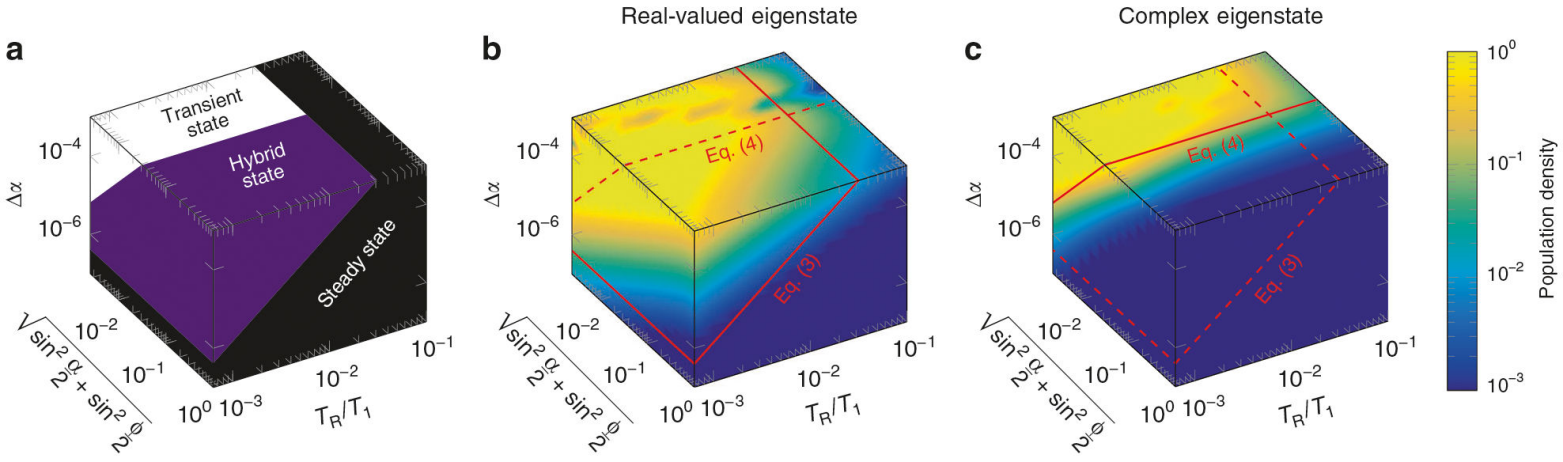
Hybrid state design space. **a** The well-known steady state is characterized by a negligible population of all (real-valued and complex) transient eigenstates and its adiabaticity condition is given by (Eq. 3): Both population densities are small whenever the change of the flip angle Δ*α* is small compared to the theoretical limit (black volume). The hybrid state described here is characterized by a population of the real-valued eigenstate while simultaneously avoiding a population of the complex-valued eigenstate. **b**, **c** In simulation, this occurs exactly in the predicted area of the parameter space (purple volume). In this volume, (Eq. 4) is fulfilled, yet (Eq. 3) is not, so that the hybrid state can occur. For the illustrative example shown here, we assumed *T*_1_ = *T*_2_, a constant Δ*α*, and Δ*ϕ* = 0. The simulation departs from the steady-state with *α* = *ϕ* and we depict the population density after 100 repetitions, each associated with a constant increase of *α* by Δ*α*

Assuming, e.g., *ϕ* = *π*, which is commonly referred to as the on-resonance condition^23,25^, this bound is at the order of one. Since the latter adiabatic condition is substantially less restrictive, the hybrid-state theory governs a vast experiment design space, as illustrated in Fig. 2.

## Adiabaticity and the solution of the Bloch equation

Hargreaves et al. showed that the eigenvector corresponding to the complex eigenvalue is approximately perpendicular to the steady-state magnetization^25^, while the real-valued eigenvalue describes the transient-state component parallel to the steady-state magnetization. By enforcing (Eq. 4), we, thus, effectively force the direction of the magnetization to adiabatically follow that of the steady states. If we then simultaneously pick our driving fields to violate (Eq. 3), the magnitude of the magnetization is in a transient state, and a hybrid of two co-existing states emerges, which we dub the hybrid state.

The adiabaticity of the magnetization’s direction effectively decouples the components of the Bloch equation, which allows us to formulate an analytic solution. For this purpose, we transform the Bloch equation into spherical coordinates and provide the solutions for the polar angle *ϑ*, the phase *φ*, and the radius *r*, which we here define as the magnitude combined with a sign (refer to the section “Methods” for the derivation). Except in the vicinity of the stop bands, which are defined by |sin *ϕ*| ≪ 1 (Supplementary Fig. 5), the polar angle can be approximated by

(5) $$\text{tan}\;\vartheta =\frac{\text{tan}\frac{\alpha }{2}}{\text{sin}\frac{\phi }{2}}$$

This equation reduces to *ϑ* = *α*/2 for *ϕ* = *π*, which we define as the on-resonance condition. In practice, *ϕ* = *π* is assigned to the on-resonant spin isochromat by the common phase increment of *π* in consecutive RF pulses. The phase of the magnetization is approximated by

(6) $$\varphi ={\text{tan}}^{-1}\left(\frac{\text{cos}\;\phi -E_{2}}{\text{sin}\;\phi }\right)-H\left\{\text{sin}\;\phi \right\}\cdot \pi +\phi_{T_{\text{E}}},$$

where the Heaviside function H disambiguates the four-quadrants and $\phi_{T_{\text{E}}}$ describes the phase of the magnetization accumulated between the RF pulse and the time the signal is observed, i.e., the echo time *T*_E_.

The radial component *r* captures the entire spin dynamics, which is described by a single first-order differential equation (Eq. (2)). This equation is solved by

(7) $$r(t)=a(t)\cdot \left(r(0)+\frac{1}{T_{1}}\int_{0}^{t}\frac{\text{cos}\;\vartheta (\tau )}{a(\tau )}\text{d}\tau \right)$$

with

$$a(\tau )=\text{exp}\left(-\int_{0}^{\tau }\frac{{\text{sin}}^{2}\;\vartheta (\xi )}{T_{2}}+\frac{{\text{cos}}^{2}\;\vartheta (\xi )}{T_{1}}\text{d}\xi \right).$$

Here, *t* denotes time and *r*(0) the initial magnetization. Alternatively, we can define the initial magnetization as a function of the final magnetization, i.e. *r*(0) = *β* · *r*(*T*_C_), where *T*_C_ denotes the duration of a single cycle of the experiment. With this boundary condition, the radial Bloch equation is solved by (Eq. 7) with

$$r(0)=\frac{\beta }{T_{1}}\frac{a\left(T_{\text{C}}\right)}{1-\beta a\left(T_{\text{C}}\right)}\int_{0}^{T_{\text{C}}}\frac{\text{cos}\;\vartheta (\tau )}{a\left(\tau \right)}\text{d}\tau .$$

When we set *β* = 1, a periodic boundary condition is obtained, which requires the magnetization at the beginning and the end of each cycle to be equal. Similarly, *β* = −1 leads to an anti-periodic boundary condition, which implies an inversion of the magnetization between cycles. Such boundary conditions enable the concatenation of multiple cycles without delays, thus, allowing for efficient signal averaging and a flexible implementation, e.g., of time-consuming 3D imaging experiments.

Intuitively, (Eq. 7) describes a predominant *T*_1_ encoding at small *ϑ*-values (close to the z-axis), and a predominant T_2_ encoding as *ϑ* approaches *π*/2, which corresponds to the *x*–*y*-plane. When *ϑ* is constant, (Eq. 7) reduces to the exponential transition into steady state described by Schmitt et al.^26^ (Supplementary Note 1). Supplementary Figure 5 validates the hybrid-state model by comparing Eqs. (5–7) to Bloch simulations for the example of anti-periodic boundary conditions.

## Robustness of the hybrid state

**Fig. 3:**
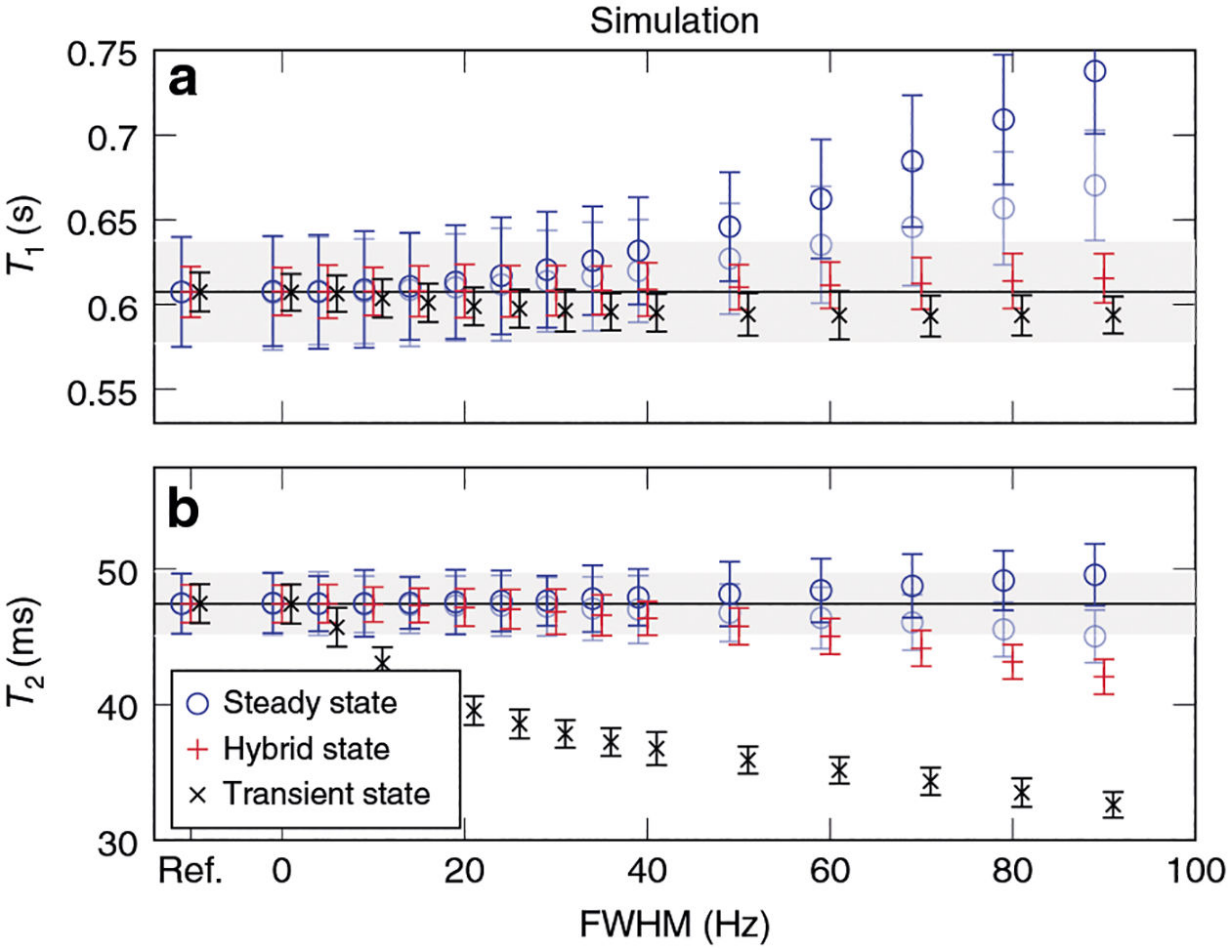
Simulation-based accuracy and precision analysis of relaxation times measurements in different spin ensemble states. The *T*_1_ relaxation times in **a** and the *T*_2_ relaxation time in **b** result from the same fit of a single simulated data set. The hybrid-state experiment is comparatively robust with respect to inhomogeneous broadening (here modeled by a Gaussian distribution of Larmor frequencies), as evident by the mean values staying within a 5% error margin (gray areas) up to a full width at half maximum (FWHM) of about 60 Hz. On the contrary, the transient state exhibits a substantial bias with increasing broadening. The steady-state experiment is robust to inhomogeneous broadening when avoiding $T_{2}^{*}$-decay in the spoiled gradient-echo segment (transparent marks). In the presence of $T_{2}^{*}$-decay, however, it also shows some systematic errors. This issue can be avoided at the cost of signal-to-noise ratio efficiency^53^. Even when using the SNR-optimal steady-state approach, the observed noise (the error bars correspond to the standard deviation) is considerably higher compared to the hybrid state. Further, the observed noise approximates the limit set by the Cramér–Rao bound well (the reference shown at the far left is the ground truth with the Cramér–Rao bound as error bars)

**Fig. 4:**
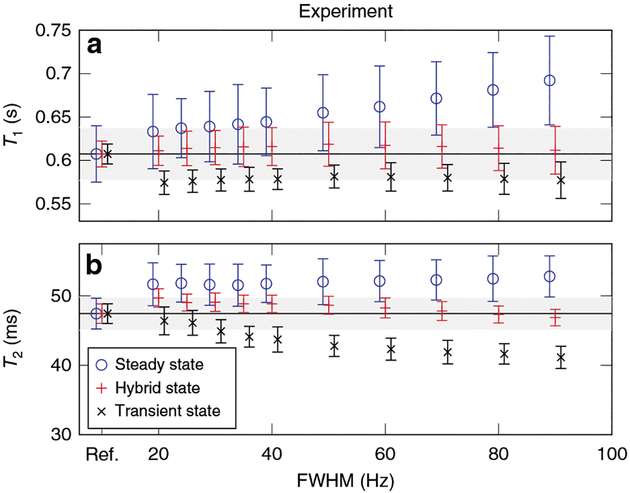
Experimental accuracy and precision analysis of relaxation times measurements in different spin ensemble states. The *T*_1_ relaxation times in **a** and the *T*_2_ relaxation time in **b** result from the same fit of a single data set. As an example of a small sphere filled with doped water, we demonstrate experimentally the benefits of the hybrid state. The mean values of the hybrid-state experiments stay well within the 5% error margin (gray areas), while the transient-state experiment reveals an increasing bias with an increasing full-width at half maximum (FWHM). Note that the shape of the Larmor-frequency distribution present in this data lacks an analytical description, making a quantitative comparison to simulations difficult. The steady state has a systematic bias, which most likely stems from the $T_{2}^{*}$-decay in the spoiled gradient-echo segment. The reference, shown at left, is composed of (inversion recovery) spin-echo experiments as the mean value and the Cramér–Rao bound as error bars

The superior robustness of the hybrid state in comparison to the fully transient state becomes evident when estimating spin relaxation times from simulated and measured signals. While the fully transient state is, in general, sensitive to deviations of both the Rabi and the Larmor frequency, inhomogeneous broadening makes latter much more difficult to correct. In order to demonstrate this, we simulated the average signal obtained from a collection of isochromats with a Gaussian distribution of Larmor frequencies and added white noise to reflect thermal noise. Because the Larmor frequency distribution in a sample is generally unknown, the obtained signals were fitted assuming a single isochromat. Figure 3 shows that the transient state leads to increasingly biased estimates of the relaxation times as the distribution of Larmor frequencies widens (full width at half maximum (FWHM) increases). Conversely, the hybrid state is more robust to inhomogeneous broadening. This finding is also validated experimentally in Fig. 4.

In most clinical imaging scenarios, the signal observed from each voxel (or volumetric pixel) is approximated well by a single Rabi frequency, which allows for easier correction^17,27^. Nevertheless, experiments that operate in the transient-state regime can be so sensitive to magnetic field variations that even small calibration errors can lead to substantial errors in the estimated relaxation times (Supplementary Note 2).

## Efficiency of the hybrid state

The simulations in Fig. 3 visualize numerically optimized experiments. Thus, we can also use them to demonstrate the superior signal-to-noise ratio (SNR) efficiency of the hybrid state in comparison to the steady state. As anticipated, the estimates retrieved from the hybrid-state experiment exhibit substantially less noise compared to the steady state. The hybrid state, thus, unites superior encoding capabilities similar to the transient state, and robustness to deviations of the magnetic fields and to inhomogeneous broadening, similar to the steady state. This finding is also validated experimentally in Fig. 4.

**Fig. 5:**
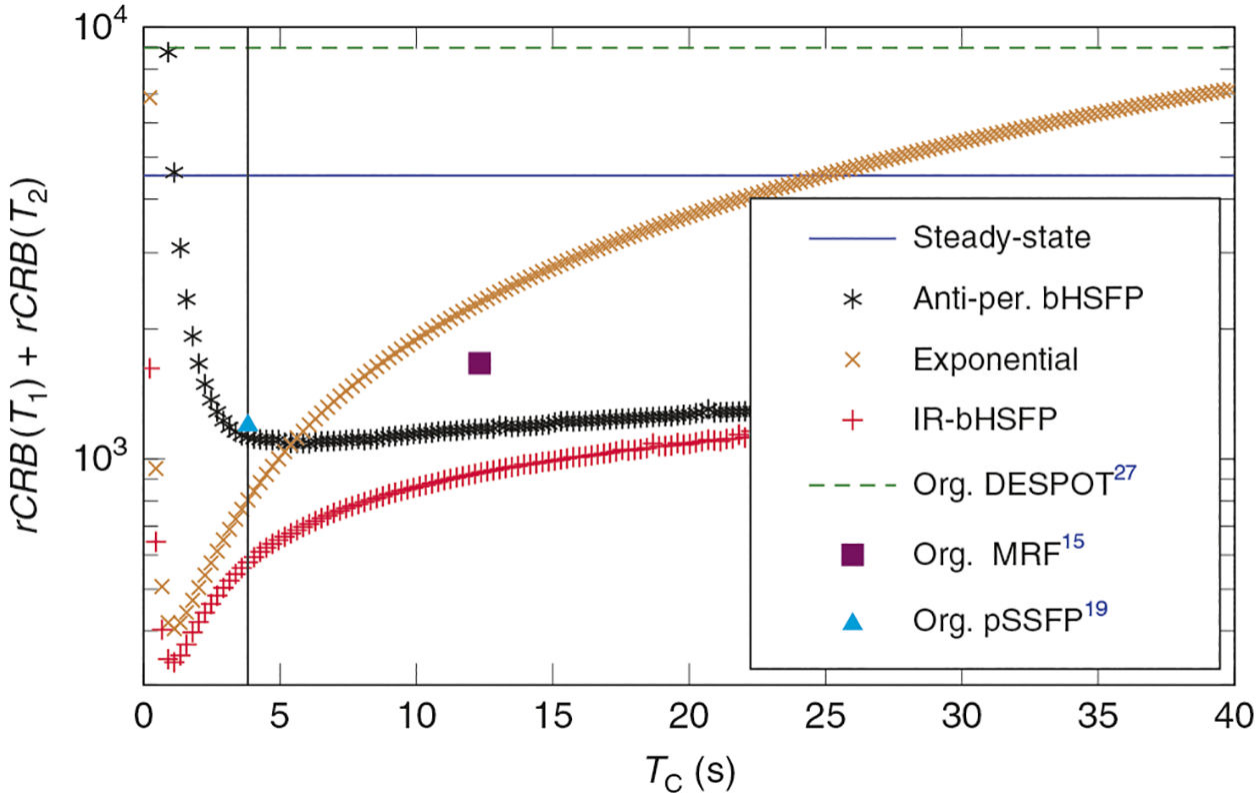
Noise analysis by virtue of the Cramér–Rao bound. The depicted relative Cramér–Rao bounds (rCRB) can be understood as a lower bound of the squared inverse signal-to-noise ratio efficiency per unit time. One can observe that, for most cycle times (*T*_C_), exponential decays as well as steady-state experiments are substantially less efficient than variants that exploit the entire experiment design space spanned by the hybrid state, namely the inversion recovery balanced hybrid-state free precession (IR-bHSFP) and the anti-periodic bHSFP experiments. For reference, some experiments from literature are shown as well, namely the original DESPOT^53^, magnetic resonance fingerprinting (MRF)^15^, and pseudo-steady-state-free precession (pSSFP)^19^ experiments. All Cramér–Rao bounds were calculated for the relaxation times *T*_1_ = 781 ms and *T*_2_ = 65 ms

For a more comprehensive analysis of the noise properties of different experiment design spaces, we examine the sum of the relative Cramér–Rao bound (rCRB) for *T*_1_-encoding and *T*_2-_encoding. The rCRB provides a lower limit for the noise in the estimated parameters, normalized by the input noise variance, by the square of the respective relaxation time and by *T*_C_/*T*_R_ (Eqs. (39) and (40)). It can be understood as a lower bound for the squared inverse SNR efficiency per unit time, and Fig. 3 shows that the simulated noise comes close to this theoretical limit. We numerically searched the parameter space of possible drive functions for the lowest combined rCRB. Due to the nature of the steady state, its rCRB does not depend on *T*_C_, so that the experiment’s duration can be chosen freely to meet the experimental needs. Hybrid-state experiments with anti-periodic boundary conditions provide a similar flexibility, since multiple cycles can be concatenated without gaps. Comparing these two experiments, one finds that the hybrid state allows for a substantially more efficient measurement than the steady state (Fig. 5).

The performance of exponential relaxation curves is here demonstrated using the example of the inversion-recovery balanced steady-state-free precession (IR-bSSFP) experiment, which is known to have a high SNR efficiency^26,28^. (Despite the name, this is actually not a steady-state experiment. Instead, one measures the magnetization as it exponentially approaches the steady state.) In contrast to the previously discussed experiments, the magnetization departs here from thermal equilibrium. This requires a long waiting time (Δ*t* ≫ *T*_1_) before the measurement can be repeated. For *T*_C_ ≲ 25 s, exponential experiments have a lower rCRB compared to steady-state experiments, and for *T*_C_ ≲ 5 s it is even lower compared to anti-periodic hybrid-state experiments (Fig. 5). An optimization of exponential experiments is essentially the search for the optimal line from the southern half of the Bloch sphere to the steady-state ellipse (Supplementary Fig. 4g). If we take the IR-bSSFP experiment and allow *ϑ*(*t*) to vary over time, we can exploit the full experiment design space spanned by the hybrid state, and we find an improved SNR-efficiency at all *T*_C_ values, with the most dramatic improvement in the case of long experiments. In analogy to the acronym IR-bSSFP, we use the term inversion-recovery balanced hybrid-state-free precession (IR-bHSFP) for hybrid-state experiments that start from thermal equilibrium by the application of an inversion pulse. We focus this analysis on experiments with balanced gradient moments because of their superior SNR properties.

In this section, we analyzed the noise properties at a single *T*_1_ and *T*_2_ value. Supplementary Note 3 demonstrates that the conclusions drawn here remain valid throughout large areas in *T*_1_–*T*_2_-space, and also in the presence of deviations of the Larmor and Rabi frequencies.

## Spin dynamics in the hybrid state

**Fig. 6:**
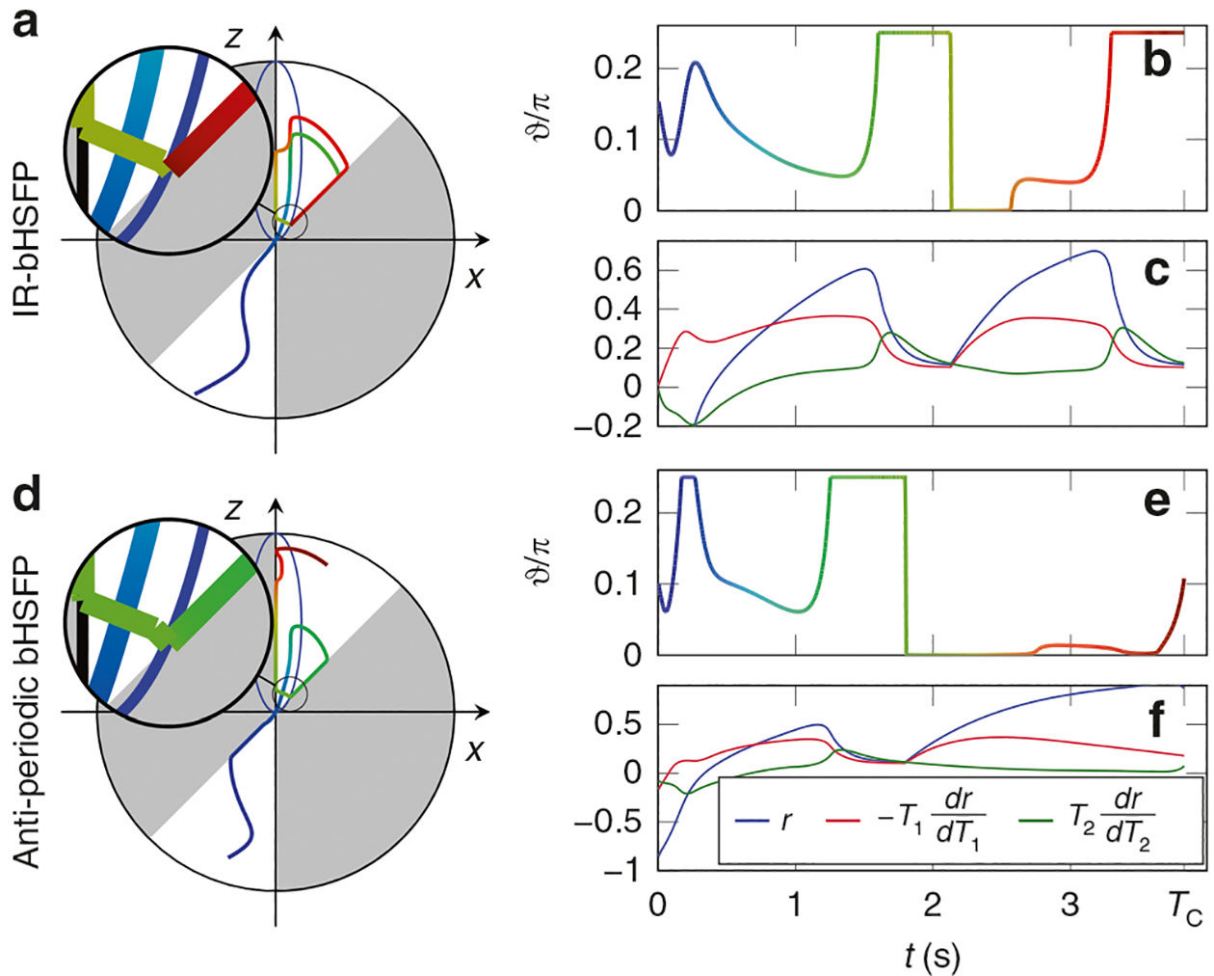
Optimized hybrid-state spin dynamics. **a** The spin dynamics in an optimized inversion recovery balanced hybrid-state free precession (IR-bHSFP) experiment on the Bloch sphere. **b** The optimized polar angle functions, with the color scale providing a reference for the trajectory on the Bloch sphere. **c** The radial component magnetization and its normalized derivatives with respect to the relaxation times, which are the foundation of computing the relative Cramér–Rao bound. **d**–**f** Repetition of **a**–**c** for an optimized bHSFP experiment with anti-periodic boundary conditions. Both experiments were jointly optimized for *T*_1_ and *T*_2_ and the polar angle was limited to 0 ≤ *ϑ* ≤ *π*/4

Optimizing the driving functions *ϑ*(*t*) results in spin trajectories with reproducible features (Fig. 6). For example, all optimizations resulted in comparatively smooth functions *ϑ*(*t*). Note that the optimizations assume a hybrid state, but otherwise do not enforce smoothness, which indicates that the adiabaticity condition (Eq. (4)) does not impair the *T*_1,2_-encoding efficiency. In some segments, the optimization exploits the design limits 0 ≤ *ϑ* ≤ *π*/4, which are imposed for practical reasons. These extreme values help to achieve a large d*r*/d*T*_1_ while minimizing d*r*/d*T*_2_ and vice versa. However, directly after crossing the origin (turquoise segment), the product of d*r*/d*T*_1_ and d*r*/d*T*_2_ has a different sign compared to the remainder of the sequence, which makes this segment valuable for decorrelating those two derivatives. As a consequence, the magnetization follows a trajectory with *ϑ* > 0. During a segment of *ϑ* ≈ 0 (yellow segment), d*r*/d*T*_2_ approaches zero. Thereafter, the optimized driving function *ϑ* increases again, resulting in non-zero signal and disentangled encoding of *r* and d*r*/d*T*_1_. Further, the optimized trajectories do not spend a significant amount of time on the steady-state ellipse. On the contrary, crossing the ellipse triggers a fast change of *ϑ*, as highlighted by the magnifications in Fig. 6.

Described hybrid-state spin trajectories result from non-convex optimizations and we can only speculate about their optimality. However, the simple and reproducible structures, together with the simple form of the governing (Eq. 2) provide an excellent basis for a more detailed analysis.

## In vivo experiment

**Fig. 7:**
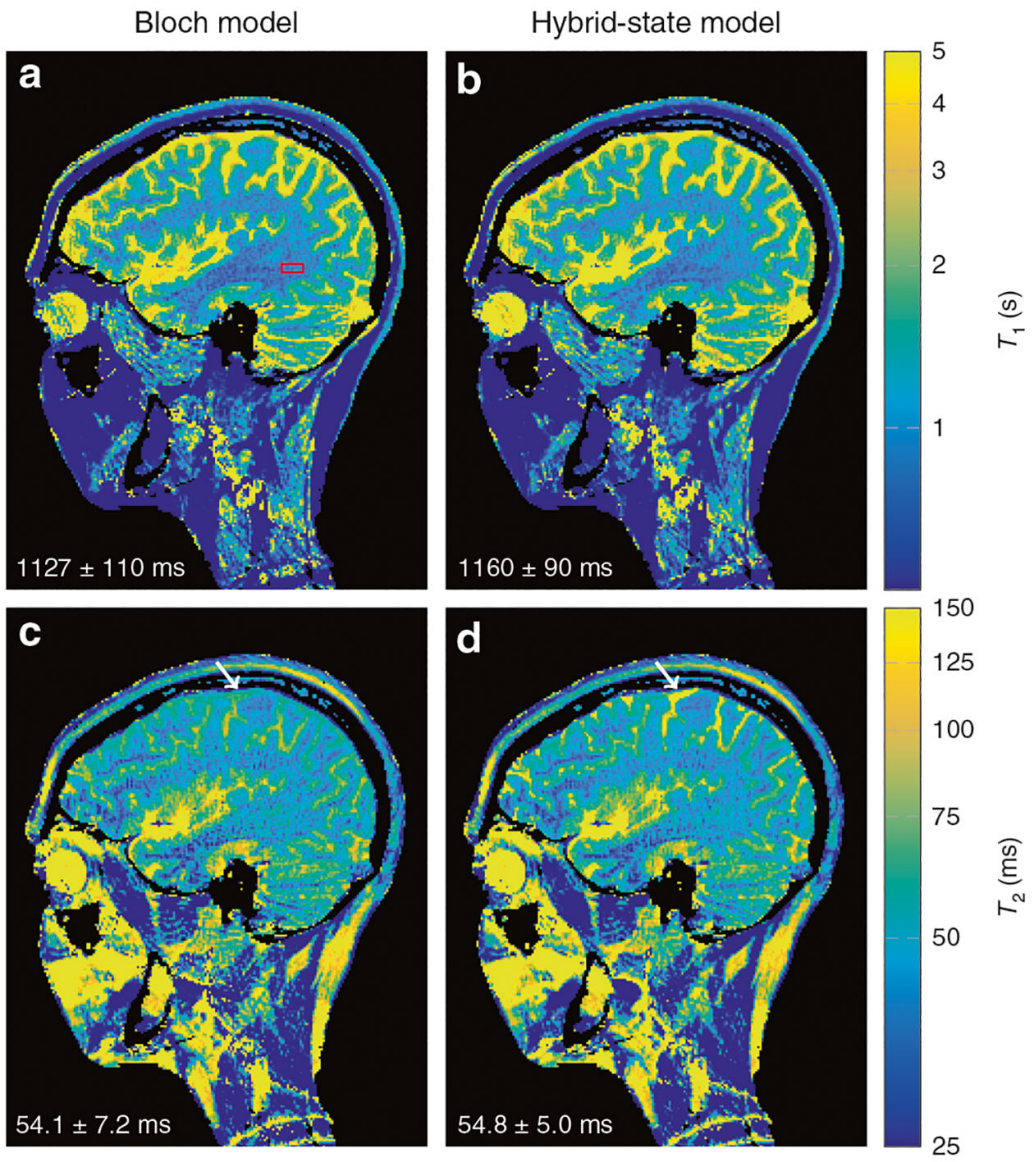
In vivo validation of the hybrid-state model. A single sagittal slice of a 3D magnetic resonance imaging experiment of a human brain is depicted. **a**, **c** The data were acquired with an anti-periodic hybrid-state experiment and were fitted with the Bloch model (Eq. (1)), and **b**, **d** with the hybrid-state model (Eqs. (5–7)). The parameter maps have a resolution of 1 mm × 1 mm × 2 mm and spatial encoding was performed with a 3D stack-of-stars *k*-space trajectory^62^. The red box indicates a region of interest used for extracting the *T*_1_ and *T*_2_ values denoted in the corner of the images. The arrows indicate a region with very long relaxation times in which slight deviations between the models can be observed. Note the logarithmic scale of the color coding

Figure 7 shows an example application of the hybrid state. The *T*_1_-map and *T*_2_-map in a sagittal slice through a human brain were acquired with an anti-periodic bHSFP experiment and demonstrate the feasibility of the hybrid state for in vivo imaging. Similar to the case of steady-state imaging, the robustness of the hybrid-state spin dynamics with respect to magnetic field variations allows us to approximate the inhomogeneously broadened spin ensemble in each voxel by a single spin isochromat with a well-defined Larmor frequency (Figs. 3 and 4). The benign response of the spin dynamics to *B*_0_ and *B*_1_ inhomogeneities—evident from the fact that Eqs. (5)–(7) are smooth functions of *α* and *ϕ* outside of the stop-band—further mitigates the propagation of unavoidable errors in estimates of those field variations, allowing for a robust correction^17,27^. Figure 7 also serves as a validation of the hybrid-state model: Fitting the data with the full Bloch model and the hybrid-state model resulted in virtually the same *T*_1_-map and *T*_2_-map, apart from regions with extremely long relaxation times (arrows in Fig. 7). This good agreement is also confirmed by the values within a region of interest comprised by white brain matter (bottom left corner in Fig. 7). All slices of this 3D acquisition can be found in Supplementary Note 4, where we also show an additional knee scan that demonstrates the robustness of hybrid-state acquisitions and alludes to their versatility.

## Scope of the hybrid-state model

Adiabatic passages are frequently used in NMR, MRI, as well as quantum computing for robust spin excitation, inversion, and refocusing in the presence of magnetic field inhomogeneities^29,30^. These passages are achieved by using continuous, slowly varying driving fields, for which the well-established adiabaticity condition $\left|\text{d}\omega_{x,y,z}/\text{d}t\right|\ll \omega_{x}^{2}+\omega_{y}^{2}+\omega_{z}^{2}$ exploits the same structure of the Hamiltonian’s spectrum as the hybrid-state condition (Eq. (4)). Further, adiabatic pulses commonly violate the corresponding steady-state condition $\left|\text{d}\omega_{x,y,z}/\text{d}t\right|\ll 1/T_{1}^{2}$ From this point of view, we can consider the hybrid state as a generalization of adiabatic passages to pulsed experiments, which allows us to exploit their robustness throughout the entire experiment. The flexible and efficient access to relaxation mechanisms, combined with the robustness of adiabatic passages constitutes the core of the hybrid-state framework.

The robustness of the measured signal to magnetic field deviations, including inhomogeneous broadening, is reflected by the hybrid-state equations of motion (Eqs. (5–7)) being smooth functions of the Larmor and Rabi frequencies, which are here parameterized by *ϕ* and *α*, respectively. This property is a direct consequence of the constrained population of the complex eigenstates and is particularly important when the line shape is unknown, e.g., when measuring biological tissue with balanced-HSFP experiments^21^. The estimation of the distribution is less problematic in unbalanced experiments, such as the fast imaging with steady-state precession^11^ (FISP) experiment, or the reversed PSIF experiment. In these experiments, one places crusher gradient pulses directly before or after the RF pulses, which essentially average the signal over *ϕ* ∈ [−2*π*, 2*π*] and desensitize the signal to inhomogeneous broadening at the cost of SNR. Assuming *ϕ* = 0 as a worst-case scenario, the hybrid-state model holds true for these experiments, and the crusher gradients can be incorporated by setting $\phi_{T_{\text{E}}}=0$ or $\phi_{T_{\text{E}}}=\phi$ in (Eq. 6) for FISP and PSIF, respectively.

For complex molecules, as well as for complex biological tissues, the Bloch equation is an oversimplified model. This can be observed in Fig. 7, where the measured relaxation times are subject to systematic deviations, which are most likely caused by magnetization transfer^31–33^. Magnetization transfer, as well as diffusion^34^ and chemical exchange^35^, are captured neither by the Bloch equation, nor by the hybrid-state model in their basic forms. However, these effects can be modeled by extensions to the hybrid-state model similarly to the established extensions of the Bloch equation^34,35^. Such extended hybrid-state models can provide a more intuitive understanding of these effects, and pave the road towards more efficient experiment designs to measure them.

## Methods

### Derivation of the hybrid state adiabaticity conditions.

#### The evolution matrix:

In order to describe pulsed MR experiments, we analyze the spin evolution matrix $U\in {\mathbb{R}}^{4\times 4}\;$, which is generated by the Hamiltonian. The matrix **U** can, e.g., be derived by taking the matrix exponential of the Hamiltonian and is not unitary due to the relaxation terms (Eq. (1)). Note that an analysis of the evolution matrix is largely equivalent to an analysis based on the Hamiltonian itself. For pulsed experiments, where we assume one hard, i.e. infinitesimally short, RF pulse surrounded by Larmor precession and relaxation, the evolution matrix is given by

(8) $$U=E\cdot R_{z}\cdot R_{y}\cdot R_{z}\cdot E,$$

where

$$E=\left(\begin{array}{cccc} \sqrt{E_{2}} & 0 & 0 & 0\\ 0 & \sqrt{E_{2}} & 0 & 0\\ 0 & 0 & \sqrt{E_{1}} & 1-\sqrt{E_{1}}\\ 0 & 0 & 0 & 1 \end{array}\right)$$

describes the relaxation of the magnetization with *E*_1,2_ = exp(−*T*_R_/*T*_1,2_). The rotation matrices

$$R_{y}=\left(\begin{array}{cccc} \text{cos}\;\alpha & 0 & -\text{sin}\;\alpha & 0\\ 0 & 1 & 0 & 0\\ \text{sin}\;\alpha & 0 & \text{cos}\;\alpha & 0\\ 0 & 0 & 0 & 1 \end{array}\right)$$

and

$$R_{z}=\left(\begin{array}{cccc} \text{cos}\;\frac{\phi }{2} & -\text{sin}\;\frac{\phi }{2} & 0 & 0\\ \text{sin}\;\frac{\phi }{2} & \text{cos}\;\frac{\phi }{2} & 0 & 0\\ 0 & 0 & 1 & 0\\ 0 & 0 & 0 & 1 \end{array}\right)$$

describe the rotations caused by the RF pulse and free precession, respectively.

Equation (8) assumes a symmetric experiment, as it is used, e.g. in balanced-SSFP experiments, where one usually measures the magnetization in the middle between two RF pulses (*T*_E_ = *T*_R_/2)^36^. In the case of unbalanced-SSFP experiments, one would usually acquire the magnetization right after each RF pulse and would place a so-called crusher gradient after the signal acquisition in order to create a net gradient moment. In such a FISP^11^ experiment, the evolution matrix would, thus, be given by **U**_FISP_ = **R**_*y*_ · **R**_*z*_ · **E**^2^ with the appropriate choice of *ϕ*, and the reversed PSIF experiment with the crusher gradient prior to the readout would be described by **U**_PSIF_ = **E**^2^ · **R**_*z*_ · **R**_*y*_. Note that derivations for FISP and PSIF lead to the same result as the one presented here.

For future reference, we also define the derivative of **U** with respect to *α*, which is given by $U^{\prime }=ER_{z}R_{y}^{\prime }R_{z}E$ with

(9) $$R_{y}^{\prime }=\left(\begin{array}{cccc} -\text{sin}\;\alpha & 0 & -\text{cos}\;\alpha & 0\\ 0 & 0 & 0 & 0\\ \text{cos}\;\alpha & 0 & -\text{sin}\;\alpha & 0\\ 0 & 0 & 0 & 0 \end{array}\right),$$

and the derivative of **U** with respect to *ϕ*, which is given by $U^{\prime }=ER_{z}^{\prime }R_{y}R_{z}E+ER_{z}R_{y}R_{z}^{\prime }E$ with

(10) $$R_{z}^{\prime }=\frac{1}{2}\left(\begin{array}{cccc} -\text{sin}\;\frac{\phi }{2} & -\text{cos}\;\frac{\phi }{2} & 0 & 0\\ \text{cos}\;\frac{\phi }{2} & -\text{sin}\;\frac{\phi }{2} & 0 & 0\\ 0 & 0 & 0 & 0\\ 0 & 0 & 0 & 0 \end{array}\right).$$

#### Eigendecomposition of the evolution matrix:

The eigendecomposition of the evolution matrix is given by

(11) $$U=V\Lambda V^{-1},$$

where $V\in {\mathbb{C}}^{4\times 4}$ is composed of the right-eigenvectors $v_{\text{d}}\in {\mathbb{C}}^{4\times 1}$ defined by Uv_d_ = *λ*_d_v_d_, and $\Lambda \in {\mathbb{C}}^{4\times 4}$ is a diagonal matrix with the eigenvalues $\lambda_{\text{d}}\in \mathbb{C}$ on the diagonal. The magnetization in MR experiments never grows arbitrarily, so that |*λ*_d_| ≤ 1 must be fulfilled for all eigenvalues. Further, if the experiment described by **U** has a non-zero steady-state magnetization, at least one eigenvalue must fulfill |*λ*_d_| = 1.

For the explicit definition of the evolution matrix in (Eq. 8), which describes one RF pulse surrounded by free precession and relaxation, one eigenvalue is given by

(12) $$\lambda_{\text{s}}=1$$

and the corresponding eigenvector describes the steady-state magnetization. As shown by Ganter^23^, the remaining eigenvalues are approximated by

(13) $$\lambda_{\|}=\frac{1}{\eta^{2}}\left({\text{cos}\;}^{2}\frac{\alpha }{2}{\text{sin}\;}^{2}\frac{\phi }{2}E_{1}+{\text{sin}\;}^{2}\frac{\alpha }{2}E_{2}\right)$$

(14) $$\lambda_{⊥}^{\left(*\right)}=\frac{{\text{e}}^{\pm i\Omega }}{2\eta^{2}}\left({\text{sin}\;}^{2}\frac{\alpha }{2}E_{1}+\left(\eta^{2}+{\text{cos}\;}^{2}\frac{\alpha }{2}{\text{sin}\;}^{2}\frac{\phi }{2}\right)E_{2}\right)$$

with

(15) $$\eta =\sqrt{{\text{cos}}^{2}\;\frac{\alpha }{2}{\text{sin}}^{2}\;\frac{\phi }{2}+{\text{sin}}^{2}\;\frac{\alpha }{2}}$$

(16) $${\text{e}}^{\pm i\Omega }=1-2\eta^{2}\pm 2\eta i\;\text{cos}\;\frac{\alpha }{2}\text{cos}\frac{\phi }{2}.$$

These eigenvalues are a first-order approximation of the parameter *δ* = (*E*_1_ − *E*_2_)/(*E*_1_ + *E*_2_), which is small for T_R_ ≪ {*T*_1_, *T*_2_} in most biological tissues^23^. The eigenvalues have an absolute value smaller than one and describe the transient state. The eigenvalue *λ*_‖_ is real-valued and the corresponding eigenvector is approximately parallel to the steady-state magnetization in the three spatial dimensions^23^. The other two eigenvalues $\lambda_{⊥}^{\left(*\right)}$ are in general complex and complex conjugate of each other, as indicated by the star. This results in the well-known oscillatory behavior of the transient state of bSSFP experiments^25^. As shown by Ganter^23^, the corresponding eigenvectors are approximately perpendicular to the steady-state eigenvector.

#### The perturbation matrix:

A sequence of *N* identical and equidistant RF pulses is simply given by **U**^*N*^ = **VΛ**^*N*^**V**^−1^ and describes the transition into the steady state^23,25^. The description of an experiment with varying driving fields, as required to avoid the steady state, is slightly more complicated. To approach this problem, we denote the evolution matrix of the *n*th repetition by **U**_*n*_ and the spin dynamics in two consecutive repetitions is described by $U_{n}U_{n-1}=V_{n}\Lambda_{n}V_{n}^{-1}V_{n-1}\Lambda_{n-1}V_{n-1}^{-1}=V_{n}\Lambda_{n}P_{n}\Lambda_{n-1}V_{n-1}^{-1}$. Here, the perturbation matrix

(17) $$P_{n}=V_{n}^{-1}V_{n-1}$$

describes the transformation from the eigenspace of **U**_*n*−1_ to the eigenspace of **U**_*n*_.

#### Expanding the perturbation matrix:

Since an explicit notation of the perturbation matrix is not very enlightening, we approximate its elements by a Taylor expansion $U_{n-1}=U\left(\kappa_{n-1}\right)=U\left(\kappa_{n}\right)-\text{Δ}\kappa_{n}U^{\prime }\left(\kappa_{n}\right)+O\left(\text{Δ}\kappa_{n}^{2}\right)$ , where $U^{\prime }\left(\kappa_{n}\right)=dU/d{\kappa |}_{\kappa =\kappa_{n}}$ denotes derivative evaluated at *κ*_*n*_. Assuming that **U**(*κ*_*n*_) is not degenerate, i.e. all eigenvalues are distinct, we can utilize the Taylor series described by Eq. (10.2) in Chapter 2 of ref.^37^ to expand the perturbation matrix (Eq. (17)). The diagonal elements are then given by *P*_*d*→*d*_ = 1 and the off-diagonal elements by

(18) $$P_{d\to f\neq d}\left(\kappa_{n},\text{Δ}\kappa_{n}\right)\approx -\frac{\text{Δ}\kappa_{n}\;\;u_{f}^{\text{H}}\left(\kappa_{n}\right)U^{\prime }\left(\kappa_{n}\right)v_{d}\left(\kappa_{n}\right)}{\left(\lambda_{d}\left(\kappa_{n}\right)-\lambda_{f}\left(\kappa_{n}\right)\right)u_{f}^{\text{H}}\left(\kappa_{n}\right)v_{f}\left(\kappa_{n}\right)},$$

where the left-eigenvectors are defined by $u_{f}^{\text{H}}\left(\kappa_{n}\right)U\left(\kappa_{n}\right)=\lambda_{f}\left(\kappa_{n}\right)u_{f}^{\text{H}}\left(\kappa_{n}\right)$ and the right-eigenvectors by **U**(*κ*_*n*_)**v**_*f*_(*κ*_*n*_) = *λ*_*f*_(*κ*_*n*_)**v**_*f*_(*κ*_*n*_). The superscript H indicates the complex conjugate transpose. Equation (18) has some similarities to the quantum mechanical adiabatic theorem^24^. In both cases, the matrix elements strongly depend on the gap between the eigenvalues. Like in the quantum mechanical case, *λ*_*S*_ – *λ*_‖_ is purely determined by the absolute value of the eigenvalues, since they both are real-valued and positive. This is fundamentally different in the case of $\lambda_{S}-\lambda_{⊥}^{\left(*\right)}$, where the gap is dominated by the complex phase of $\lambda_{⊥}^{\left(*\right)}$. In the following, we will show that this key difference opens the door for?the hybrid state to emerge.

#### The population of the transient eigenstates:

In order to analyze the cumulative population transfer during *N* repetitions, we describe the corresponding spin dynamics by

(19) $$\prod_{n=1}^{N}U_{N-n}=V_{N-1}\left(\prod_{n=1}^{N-1}\Lambda_{N-n}P_{N-n}\right)\Lambda_{0}V_{0}^{-1}.$$

The goal of this section is to extract the essential elements of this matrix product and to derive boundary conditions for avoiding a population of the individual eigenstates that describe the transient state magnetization. For this purpose, we will first show that only the population transfer from the steady state is of relevance.

The steady-state left-eigenvector becomes evident to be $u_{\text{S}}^{H}=(0,0,0,1)$ by multiplying it from the left to **U** (Eq. (8)). For either parameter variation, we obtain $u_{\text{S}}^{H}U^{\prime }=(0,0,0,0)$ since the last rows of $R_{y}^{\prime }$ and $R_{z}^{\prime }$ contain only zeros (Eqs. (9) and (10)). With (Eq. 18), it follows that *P*_*d*→*S*_ = 0∀*d ≠ S*, resulting in the following structure of the perturbation matrix:

$$P_{n}\approx \left(\begin{array}{cccc} 1 & 0 & 0 & 0\\ P_{\text{S}\to \|}\left(\kappa_{n},\Delta \kappa_{n}\right)+O\left(\Delta \kappa_{n}^{2}\right) & 1 & O\left(\Delta \kappa_{n}\right) & O\left(\Delta \kappa_{n}\right)\\ P_{\text{S}\to ⊥}\left(\kappa_{n},\Delta \kappa_{n}\right)+O\left(\Delta \kappa_{n}^{2}\right) & O\left(\Delta \kappa_{n}\right) & 1 & O\left(\Delta \kappa_{n}\right)\\ P_{\text{S}\to ⊥}^{*}\left(\kappa_{n},\Delta \kappa_{n}\right)+O\left(\Delta \kappa_{n}^{2}\right) & O\left(\Delta \kappa_{n}\right) & O\left(\Delta \kappa_{n}\right) & 1 \end{array}\right)$$

Here, only the essential elements are denoted explicitly. The central part of (Eq. 19) describes the combined effect of *N* RF pulses with varying parameters onto the eigenvectors and is given by

(20) $$\prod_{n=0}^{N-1}\Lambda_{N-n}P_{N-n}\approx \left(\begin{array}{cccc} 1 & 0 & 0 & 0\\ \sum_{n=1}^{N}P_{S-\|}\left(\kappa_{n},\Delta \kappa_{n}\right)\prod_{k=n}^{N}\lambda_{\|}\left(\kappa_{k}\right)+O\left(\Delta \kappa_{n}^{2}\right) & O\left(\lambda^{N}\right) & O\left(\Delta \kappa \cdot \lambda^{N}\right) & O\left(\Delta \kappa \cdot \lambda^{N}\right)\\ \sum_{n=1}^{N}P_{S\to ⊥}\left(\kappa_{n},\Delta \kappa_{n}\right)\prod_{k=n}^{N}\lambda_{⊥}\left(\kappa_{k}\right)+O\left(\Delta \kappa_{n}^{2}\right) & O\left(\Delta \kappa \cdot \lambda^{N}\right) & O\left(\lambda^{N}\right) & O\left(\Delta \kappa \cdot \lambda^{N}\right)\\ \sum_{n=1}^{N}P_{S\to ⊥}^{*}\left(\kappa_{n},\Delta \kappa_{n}\right)\prod_{k=n}^{N}\lambda_{⊥}^{*}\left(\kappa_{k}\right)+O\left(\Delta \kappa_{n}^{2}\right) & O\left(\Delta \kappa \cdot \lambda^{N}\right) & O\left(\Delta \kappa \cdot \lambda^{N}\right) & O\left(\lambda^{N}\right) \end{array}\right).$$

For the leading order error term, the differences between the three different $\lambda_{\|,⊥}^{\left(*\right)}$ and the dependency on the experimental parameters are neglected, and the product of any combination of eigenvalues is denoted by *λ*^*N*^. Equation (20) shows that all matrix elements except the first column approach zero for large *N* since $\left|\lambda_{\|,⊥}^{\left(*\right)}\right|<1$. This reveals that the population transfer between the individual transient eigenstates are negligible, and we are left with the population transfer from the steady eigenstate to the transient eigenstates, as described by the first column. Its entries describe the counteraction of populating the transient eigenstates, denoted by *P*_S→*f*_(*κ*_*n*_, Δ*κ*_*n*_) with *f* ∈ {‖, ⊥, ⊥^✶^}, and the relaxation of the transient eigenstates in the time span between their population and the time of observation after *N* repetitions, denoted by $\prod_{k=n}^{N}\lambda_{f}\left(\kappa_{k}\right)$.

Our goal here is to understand how slowly we should drive the system to maintain negligible population transfer from the steady to the transient eigenstates. In order to assess how many terms in (Eq. 20) are relevant, we calculate the following limit:

(21) $$\left|\prod_{k=n}^{N}\lambda_{f}\left(\kappa_{k}\right)\right|\leq \underset{k}{\text{max}}{\left|\lambda_{f}\left(\kappa_{k}\right)\right|}^{N-n}≲{\left(1-\epsilon \right)}^{N-n},$$

with

(22) $$\epsilon =1-E_{2}\approx \frac{T_{\text{R}}}{T_{2}}.$$

Therefore, we can neglect all summands that fulfill

(23) $$N-n\gg \epsilon^{-1}.$$

Assuming that we change experimental parameters slowly over this time span, we can use the Taylor expansion

(24) $$\lambda_{f}\left(\kappa_{n}\right)\approx \lambda_{f}\left(\kappa_{N}\right)-(N-n)\Delta \kappa_{N}\frac{\partial \lambda_{f}\left(\kappa_{N}\right)}{\partial \kappa_{N}}$$

with

(25) $$\left|\frac{\partial \lambda_{f}\left(\kappa_{N}\right)}{\partial \kappa_{N}}\right|\leq 1.$$

Equation (25) becomes evident from Eqs. (13) and (14). Equation (18) approximates the elements of the perturbation matrix to the first order of Δ*κ*_*n*_. In this approximation, *P*_S→*f*_(*κ*_*n*_, Δ*κ*_*n*_) is constant and we can approximate

(26) $$\sum_{n=1}^{N}P_{\text{s}-f}\left(\kappa_{n},\Delta \kappa_{n}\right)\prod_{k=n}^{N}\lambda_{f}\left(\kappa_{k}\right)\approx P_{\text{S}\to f}\left(\kappa_{N},\Delta \kappa_{N}\right)\;\sum_{n=1}^{N}\left(\lambda_{N}^{N+1-n}+\lambda_{N}^{N-n}\frac{\Delta \kappa_{N}}{2}(N-n)(N+1-n)\frac{\partial \lambda_{f}\left(\kappa_{N}\right)}{\partial \kappa_{N}}\right)\;\approx \frac{P_{\text{s}-f}\left(\kappa_{N},\Delta \kappa_{N}\right)}{1-\lambda_{f}\left(\kappa_{N}\right)}\left(1+\frac{3\Delta \kappa_{N}\lambda_{N}}{{\left(1-\lambda_{N}\right)}^{2}}\frac{\partial \lambda_{f}\left(\kappa_{N}\right)}{\partial \kappa_{N}}\right)$$

by employing the geometric series.

In order to derive a limit under which we can neglect the individual transient eigenstates, we compare the corresponding elements of the first column in (Eq. 20) to the element corresponding to the steady-state eigenstate, which is unity. Examining (Eq. 26), we find that we can neglect the last term in the brackets as long as

(27) $$\left|\Delta \kappa_{N}\right|\ll {\left|1-\lambda_{N}\right|}^{2}.$$

By doing so, we find the condition

(28) $$\underset{n}{\text{max}}\frac{\left|P_{S\to f}\left(\kappa_{n},\Delta \kappa_{n}\right)\right|}{\left|1-\lambda_{f}\left(\kappa_{n}\right)\right|}\ll 1,$$

which ensures that the corresponding eigenstate is not populated.

It will turn out (see the section “The hybrid state adiabaticity condition”) that the Condition (27) is equivalent to the Condition (28), being the necessary adiabaticity condition to remain in the hybrid state. We also note that accounting for the higher-order Taylor expansion terms ((Δ*κ*_*n*_)^*m*^ with *m* = 2, 3, …) in Eqs. (26) and (28), does not qualitatively change our adiabaticity bound (Eq. (27)). Indeed, after the summation, such higher-order terms result in the contributions where each extra power of Δ*κ*_*n*_ in the denominator (from the higher order approximations of the perturbation matrix elements, Eq. (11.3) in Chapter 2 of ref.^37^), is compensated by the extra power of order |1 − *λ*_*f*_(*κ*_*n*_)| in the denominator (arising from the corresponding sums such as $\sum_{n}n\lambda^{n}\approx 1/{\left(1-\lambda \right)}^{2}$ and so on). Hence, as long as |Δ*κ*_*n*_| ≪ |1 − *λ*_*f*_(*κ*_*n*_)|, which is automatically satisfied due to Condition (27) from the leading-order term, taking into account the higher-order terms in the Taylor expansion would exceed the accuracy for our approximation. Thus, (Eq. 28) is the most stringent bound.

#### The hybrid state adiabaticity condition:

In this section, we will use the Taylor expansion in (Eq. 18) to solve (Eq. 28) for the cases of the perpendicular eigenstates, i.e. for *f* = ⊥(*). Note that *P*_S→⊥_ and $P_{S\to ⊥}^{*}$, as defined by (Eq. 18), are complex conjugate of each other.

Assuming that the eigenvectors are normalized to have a unit *ℓ*_2_-norm, we can bound the numerator of (Eq. 18) by

(29) $$\left|u_{f}^{\text{H}}\left(\kappa_{n}\right)U^{\prime }\left(\kappa_{n}\right)v_{d}\left(\kappa_{n}\right)\right|\leq {\left\|U^{\prime }\right\|}_{2}\leq 1.$$

The here employed subordinate matrix norm is given by the square root of the largest eigenvalue of (**U**′)^H^**U**′ and is smaller than one since the **U**′ consists only of rotations and relaxation terms (Eq. (53.5), Chapter 1 and Eq. (8.4), Chapter 2 of ref.^37^).

The first term of the denominator in (Eq. 18), $1-\lambda_{⊥}^{\left(*\right)}$, describes the gap of the eigenvalues. We can assume $\left|\lambda_{⊥}^{\left(*\right)}\right|=1$ as a worst case scenario and bound this gap the complex phase Ω. This gap can only be small when Ω approaches zero (Eqs. (14–16)), so that we can use a Taylor expansion of (Eq. 16)

(30) $$\text{Im}{\left\{{\tilde{\lambda }}_{⊥}^{\left(*\right)}\right\}}^{2}\approx 4{\text{Ω}}^{2}=4\left({\text{sin}}^{2}\;\frac{\alpha }{2}+{\text{sin}}^{2}\;\frac{\phi }{2}\right)$$

to derive the limit

(31) $$\left|1-\lambda_{⊥}^{\left(*\right)}\right|\geq 2\Omega$$

The last term in (Eq. 18) that requires our attention is $u_{⊥}^{\text{H}}v_{⊥}$. In order to assess the scenarios under which this product is small, we can approximate the evolution matrix by $U\approx R+\frac{\epsilon }{2}D+O\left(\epsilon^{2}\right)$, which describes it as a small perturbation of the unitary rotation matrix **R** = **R**_*z*_**R**_*y*_**R**_*z*_. The perturbation is of the order *ϵ* (Eq. (22)), and **D** = {**R**, **C**} is the anti-commutator of the rotation matrix and

$$C=\left(\begin{array}{cccc} -1 & 0 & 0 & 0\\ 0 & -1 & 0 & 0\\ 0 & 0 & -1 & 1\\ 0 & 0 & 0 & 0 \end{array}\right).$$

which approximates the relaxation matrix by $E\approx 1+\frac{\epsilon }{2}C$ when assuming *δ* ≪ 1. In this perturbation picture, the product of left-eigenvector and right-eigenvector $u_{f}^{\text{H}}v_{f}$ of the evolution matrix is approximated by

(32) $$u_{f}^{\text{H}}v_{f}\approx 1+\frac{\epsilon^{2}}{4}\sum_{d\neq f}\frac{\left({\tilde{v}}_{d}^{\text{H}}D\;{\tilde{v}}_{f}\right)\left({\tilde{v}}_{f}^{\text{H}}D\;{\tilde{v}}_{d}\right)}{{\left({\tilde{\lambda }}_{f}-{\tilde{\lambda }}_{d}\right)}^{2}},$$

where the tilde indicates the eigenvalues and vectors of **R** (Eq. (18) or Eq. (10.2) in Chapter 2 of ref.^37^). The first term results from the property ${\tilde{u}}_{f}^{\text{H}}{\tilde{v}}_{f}=1$ of the eigenvectors of **R**. Due to the orthornormality of the eigenspace of **R**, we further eliminated the terms that are linear in *ϵ*. With the bound ||D||_2_ ≤ 1 and the normalization of the eigenvectors, we obtain $\left|{\tilde{v}}_{d}^{\text{H}}D{\tilde{v}}_{f}\right|\leq 1$. Further, we can derive the eigenvalues of **R** from Eqs. (13) and (14) by setting *E*_1_ = *E*_2_ = 1 and find ${\tilde{\lambda }}_{S}={\tilde{\lambda }}_{\|}=1$ and ${\tilde{\lambda }}_{⊥}^{\left(*\right)}={\text{e}}^{\pm i\Omega }$.

We adopt the bound in (Eq. 31) for *d* ϵ {S, ‖}; and for *d* = ⊥*we find ${\left|{\tilde{\lambda }}_{⊥}-{\tilde{\lambda }}_{⊥}^{*}\right|}^{2}\geq 8\Omega^{2}$. This bound neglects the scenario in which $\lambda_{⊥}^{\left(*\right)}$ both approach negative one, which is the case when $\left|\text{cos}\frac{\alpha }{2}\right|\ll 1\text{ or }\left|\text{cos}\frac{\phi }{2}\right|\ll 1$. Note that this leads to a breakdown of the approximations made for deriving (Eq. 14). Since both eigenvalues have the same complex phase, we can treat those two components jointly and without proof we state that both scenarios result in ${\left\|P_{S\to ⊥}^{\left(1\right)}v_{⊥}^{\left(1\right)}+P_{S\to ⊥}^{\left(2\right)}v_{⊥}^{\left(2\right)}\right\|}_{2}\ll 1$, where the superscript indicates the two formally complex conjugate components. In other words, when the eigenvalues $\lambda_{⊥}^{\left(*\right)}$ approach negative one, the perpendicular eigenstates are not populated.

By summing over all three terms, we arrive at

(33) $$\left|u_{⊥}^{\text{H}}v_{⊥}\right|\geq 1-\frac{9}{32}\frac{\epsilon^{2}}{\Omega^{2}}.$$

A hybrid state only occurs if *ϵ* ≪ Ω. Therefore, we can neglect the second term when deriving the hybrid-state condition. Inserting the bounds of the individual terms of the perturbation matrix (Eqs. (29), (31), and (33)) into (Eq. 18), we find

(34) $$\left|P_{S\to ⊥}^{\left(*\right)}\left(\alpha_{n},\phi_{n},\Delta \kappa_{n}\right)\right|\leq \frac{\Delta \kappa_{n}}{2\Omega }.$$

This bound describes how much magnetization is at most transfered from the steady state to the orthogonal eigenstates by varying *α* or *ϕ* between two consecutive repetitions.

Further, inserting (Eq. 34) into (Eq. 28) in order to account for the cumulative population, and utilizing (Eq. 31), we arrive at the limit

(4a) $$\underset{n}{\text{max}}\left|\Delta \kappa_{n}\right|\ll 8\left({\text{sin}}^{2}\;\frac{\alpha_{n}}{2}+{\text{sin}}^{2}\;\frac{\phi_{n}}{2}\right).$$

When this adiabaticity condition is fulfilled, we can neglect the perpendicular transient eigenstates. For simplicity, we can drop the factor of 8 in (Eq. 4).

#### The steady-state adiabaticity condition:

In order to do the same analysis for the parallel transient eigenstate, we have to rely on the absolute value of *λ*_‖_, since it is real-valued and positive. Note that $u_{\|}^{\text{H}}v_{\|}$ cannot be bound in the same way as done in (Eq. 33), since the eigenvalues ${\tilde{\lambda }}_{S}={\tilde{\lambda }}_{\|}$ are degenerate. Since the steady-state adiabaticity condition is not essential for this work, we skip the degenerate perturbation theory and assume $u_{\|}^{\text{H}}v_{\|}\approx 1$. With the bound *λ*_‖_ ≤ *E*_1_, which results from (Eq. 13), and with Eqs. (28) and (29), we arrive at the adiabaticity condition

(3a) $$\left|\Delta \kappa_{n}\right|\ll {\left(1-E_{1}\right)}^{2}\approx {\left(T_{\text{R}}/T_{1}\right)}^{2},$$

which ensures that the parallel transient state is negligible.

### The Bloch equation in spherical coordinates.

Under the derived adiabaticity condition, the hybrid state emerges, and we observe transient-state behavior only along the direction of the steady-state magnetization. Transforming the Bloch equation into spherical coordinates isolates the transient-state behavior in a single dimension, and the components of the Bloch equation uncouple into first-order differential equations that can be solved.

Spherical coordinates are here defined by *x* = *r* sin *ϑ* cos *φ*, *y* = *r* sin *ϑ* sin *φ* and *z* = *r* cos *ϑ*, where *r* is the radius, *ϑ* the polar angle or the angle between the magnetization and the *z*-axis and *φ* is the azimuth or the angle between the *x*-axis and the projection of the magnetization onto the *x*–*y* plane. For practical reasons, we use the limits −1 ≤ *r* ≤ 1, 0 ≤ *ϑ* ≤ *π*/2, and 0 ≤ *φ* < 2*π* to uniquely identify the polar coordinates. Thermal equilibrium is given by *r*_0_ = 1, *ϑ*_0_ = 0, and *φ*_0_ = 0, where the latter can be chosen freely.

Since the azimuth, or phase, adiabatically follows the steady state, we can transform the known Cartesian steady-state solutions (Eqs. (6) and (7) in ref.^12^) to spherical coordinates, which results in (Eq. 6). The polar angle can be derived from Eqs. (9)–(11) in ref.^12^ and is given by

(35) $$\text{tan}\;\vartheta =\frac{\sqrt{E_{2}}\;\text{sin}\;\alpha \sqrt{1-2E_{2}\;\text{cos}\;\phi +E_{2}^{2}}}{G+\sqrt{E_{1}}\left(E_{2}-\text{cos}\;\phi \right)+\left(1-E_{2}\;\text{cos}\;\phi \right)\text{cos}\alpha )}$$

with

$$G=\frac{\left(1-E_{1}\;\text{cos}\;\alpha \right)\left(1-E_{2}\;\text{cos}\;\phi \right)-\left(E_{1}-\text{cos}\;\alpha \right)\left(E_{2}-\text{cos}\;\phi \right)E_{2}}{1+\sqrt{E_{1}}}.$$

With a Taylor expansion at *E*_2_ = 1, the polar angle is described by

(36) $${\text{sin}}^{2}\;\vartheta =\frac{{\text{sin}}^{\text{2}}\frac{\alpha }{2}}{{\text{sin}}^{2}\frac{\phi }{2}\cdot {\text{cos}}^{\text{2}}\frac{\alpha }{2}+{\text{sin}}^{\text{2}}\frac{\alpha }{2}}+\left(1-E_{2}\right)\cdot \xi +O\left({\left(1-E_{2}\right)}^{2}\right)$$

with

$$\xi =\frac{4{\left(\text{cos}\;\alpha -1\right)}^{2}\left(\sqrt{E_{1}}-1\right)}{\left(\sqrt{E_{1}}+1\right){\left(\text{cos}\;\alpha +\text{cos}\;\phi +\text{cos}\;\alpha \;\text{cos}\;\phi -3\right)}^{2}}.$$

The factor *ξ* is only large, if cos *ϕ* ≈ (3 − cos *α*)/(cos *α* + 1), which is only the case, if |1 − cos *α*| ≪ 1 and |1 − cos *ϕ*| ≪ 1 are simultaneously fulfilled, i.e. for small flip angles in the vicinity of the stop-band. Consequently, for standard imaging scenarios with *T*_R_ ≪ *T*_2_ the polar angle can be approximated by (Eq. 5) apart from the vicinity of the stop band.

The spherical coordinate *r* captures the transient-state spin dynamics, and we can derive (Eq. 2) simply by transforming the Bloch equation into spherical coordinates^14,38^.

#### B_1_-inhomogeneities:

One can describe the effect of *B*_1_-inhomogeneities on the spins by $\alpha =B_{1}/B_{1}^{\text{nom}.}\alpha^{\text{nom.}}$, where $B_{1}^{\text{nom.}}$ and *α*^nom.^ describe the nominal *B*_1_-field and flip angle, respectively. The effect on the polar angle is described by inserting this relation into (Eq. 5) and successively into (Eq. 7).

In order to implement anti-periodic boundary conditions, the magnetization must be inverted between successive cycles (*r*(0) = −*r*(*T*_C_)), while changes of *ϑ* and *ϕ* are required to remain within limits in order not to violate the adiabaticity condition posed in (Eq. 4a). Applying a *π*-pulse with an inhomogeneous *B*_1_-field would lead to severe fluctuations of *ϑ*, causing a violation of the adiabaticity condition. In order to mitigate these fluctuations, we surround the inversion pulse by crusher gradients. As shown in refs.^39,40^, the transversal magnetization *M*_⊥_ refocuses after inversion pulse with crusher gradients to an echo of the size $M_{⊥}^{+}={\text{sin}}^{2}\left(\pi /2\cdot B_{1}/B_{1}^{\text{nom}}\cdot \right)M_{⊥}^{-}$, where the superscript + and − indicate the magnetization before and after the RF pulse, respectively. The longitudinal magnetization, on the other hand, is given by $M_{z}^{+}=\text{cos}\left(\pi B_{1}/B_{1}^{\text{nom}.}\right)M_{z}^{-}$. In spherical coordinates, this leads to

(37) $$\text{tan}\;\vartheta^{+}=\frac{{\text{sin}}^{2}\left(\frac{\pi }{2}\frac{B_{1}}{B_{1}^{\text{nom.}}}\right)}{\text{cos}\left(\pi \frac{B_{1}}{B_{1}^{\text{nom.}}}\right)}\text{tan}\;\vartheta^{-}.$$

In the human brain at 3T, one usually observes variations in the range of $B_{1}/B_{1}^{\text{nom.}}\in [0.8,1.2]$^41^. Within this range, the resulting effect is bound by |*ϑ*^+^/*ϑ*− − 1| < 0.12 and will be neglected in the following.

In return, the crusher gradients manipulate *r*, which is accounted for by setting

(38) $$\beta =-\sqrt{{\text{sin}}^{2}\;\vartheta^{-}\cdot {\text{sin}}^{4}\frac{\pi B_{1}}{2B_{1}^{\text{nom.}}}+{\text{cos}}^{2}\;\vartheta^{-}\cdot {\text{cos}}^{2}\frac{\pi B_{1}}{B_{1}^{\text{nom.}}}}$$

in (Eq. 7). Repeating the inversion pulses with the same spoiling gradients can potentially result in higher order spin echoes and stimulated echoes, impairing the derived description of the spin physics. However, when using *T*_C_ ≫ *T*_2_, we can assume that those contributions are negligible.

### Numerical optimizations.

#### Cramér–Rao bound:

The Cramér–Rao bound^42,43^ provides a universal limit for the noise variance of a measured parameter, given that the reconstruction algorithm is an unbiased estimator. This very general and established metric has been utilized for optimizing MR parameter mapping experiments in refs.^44–46^ amongst others, and to MRF in ref.^47^. In discretized notation, the Cramér–Rao bound is defined by the inverse of the Fisher information matrix **F** with the entries $F_{ij}=b_{i}^{\text{T}}b_{i}/\sigma^{2}$ given by

$$b_{1}=\text{d}x/\text{d}M_{0}$$

$$b_{2}=\text{d}x/\text{d}T_{1}$$

$$b_{3}=\text{d}x/\text{d}T_{2}.$$

Here $x\in {\mathbb{R}}^{N_{t}}$ is a vector describing the measured signal or, equivalently, the transversal magnetization at *N*_*t*_ discrete time points, and *σ*^2^ is the input variance. Each element of the vector is given by *x*_*n*_ = *M*_0_*r*(*t*_*n*_)·sin *ϑ*(*t*_*n*_). The vectors **b**_*i*_ describe the derivatives of the signal evolution with respect to all considered parameters. For the optimizations, we normalize the proton density to *M*_0_ = 1, so that **b**_1_ = **x**.

In this work, we focused on quantifying relaxation times, since a measured *M*_0_, as defined in this work, is modulated by the receive coil sensitivity and provides only a relative measure. We can define the dimensionless rCRB to be

(39) $$\text{rCRB}\left(T_{1}\right)=\frac{1}{\sigma^{2}T_{1}^{2}}\frac{T_{\text{C}}}{T_{\text{R}}}{\left(F^{-1}\right)}_{2,2}$$

(40) $$\text{rCRB}\left(T_{2}\right)=\frac{1}{\sigma^{2}T_{2}^{2}}\frac{T_{C}}{T_{R}}{\left(F^{-1}\right)}_{3,3}.$$

The normalization by the variances cancels out the variance in the definition of the Fisher information matrix, and the normalization by the relaxation time is done to best reflect the *T*_1,2_-to-noise ratio (defined as $T_{1,2}/\sigma_{T_{1,2}}$). Further, the multiplication with *T*_C_/*T*_R_ normalizes the rCRB by duration of the experiment such that it can be understood as the squared inverse SNR efficiency per unit time, given a fixed *T*_R_.

#### Optimal control:

The polar angle *ϑ* is here treated as the control parameter for spin dynamics along the radial direction as by (Eq. 2). Thus, we can employ the rich optimal control literature^48,49^ for numerical optimization of *ϑ*(*t*). We used a Broyden–Fletcher–Goldfarb–Shanno (BFGS) algorithm^50^ with rCRB(*T*_1_) + rCRB (*T*_2_) as an objective function. To further improve convergence, the BFGS algorithm is embedded in a scatter search algorithm which tried 1000 starting points^51^. The numerical optimization was based on *ϑ*(Δ*t* · *n*) with a discrete step size of Δ*t* = 4.5 ms and the evaluation points *n* ∈ {1, 2, …, *T*_C_/Δt}. The gradient of the objective function with respect to *T*_1_, *T*_2_, and each *ϑ*(Δ*t* · *n*) was explicitly calculated.

Since the rCRB intrinsically compares a signal evolution to its surrounding in the parameter space, only a single set of relaxation times is necessary for the optimization. Here, we used the relaxation times *T*_1_ = 781 ms and *T*_2_ = 65 ms, corresponding to the values measured for white matter as reported in ref.^16^. All optimizations were initialized with the pattern provided in the pseudo-SSFP paper^19^ and the optimizations were performed with the constraint 0 ≤ *ϑ* ≤ *π*/4, which limits the flip angle to *α* ≤ *π*/2, ensuring consistent slice profiles by virtue of the linearity in the small tip-angle approximation^52^, and aiding compliance with safety considerations by avoiding high power large flip-angle pulses.

### Phantom simulations.

In order to visualize the noise properties of the transient-state, the hybrid-state, and the steady-state, as well as the systematic errors arising from inhomogeneous broadening, we simulated the signal generated by a spin ensemble with a Gaussian distribution of Larmor frequencies, added Gaussian noise and performed a non-linear least-square fit.

The hybrid-state is exemplified by the optimized sequence with anti-periodic boundary conditions (Fig. 6d–f), the transient-state by the original MRF-pattern^15^ (Supplementary Fig. 4a–d) modified to have the same constant *T*_R_ = 4.5 ms as the other two sequences, and the steady-state by the Cramér–Rao-bound optimized sequence depicted in Supplementary Fig. 4m–o. Note that the optimization resulted in one distinct flip angle for the spoiled gradient-recalled echo (SPGR) segment and two distinct flip angles for the bSSFP segment. We use this pattern in order to provide an upper bound of the steady-state’s SNR performance even though it is sensitive to $T_{2}^{*}$-decay in the SPGR segment. For this reason, we performed simulations that neglect and account for $T_{2}^{*}$-decay, respectively. Note that the experiment can be desensitized to $T_{2}^{*}$-decay by using two SPGR segments with distinct flip angles, which comes, however, at the cost of SNR efficiency^53^.

### Phantom experiments.

In order to experimentally demonstrate the benefits of the hybrid state, we measured relaxation times in a homogeneous, spherical, table-tennis ball-sized phantom filled with doped water on a clinical 3T Prisma scanner (Siemens, Erlangen, Germany). We used a commercial transmit-receive knee RF-coil for excitation and one of its 15 elements for signal reception.

For reference, we measured *T*_1_ with inversion recovery spin-echo experiments with different inversion times and fitting an exponential function. Similarly, we performed multiple spin-echo experiments with different echo times in order to measure a *T*_2_ reference.

With each of the three considered excitation patterns (see the section “Phantom simulations”), we performed experiments without any spatial encoding. At the beginning of the transient-state MRF sequence, a secant inversion pulse with a duration of 10.24 ms was applied followed by a spoiler gradient. Thereafter, the sequence consists only of RF pulses alternating with signal reception. The RF pulses were implemented as 1000 μs long rectangular pulses following the flip angle schemes shown in Supplementary Fig. 4b and using *T*_R_ = 4.5 ms. Since this sequence starts from thermal equilibrium, consecutive repetitions were separated by a 10 s pause. The anti-periodic boundary conditions used for the hybrid-state experiment allows for consecutive repetitions were acquired without any gaps. We use a rectangular *π*-pulse surrounded by crusher gradients for inverting the magnetization between consecutive repetitions. The steady-state experiments require a transition phase in order to reach the steady-state. Thereafter, all repetitions for each segment were acquired without gaps.

Each repetition was fitted with a non-linear least-square fit. The fitted function accounts for the finite pulse duration by approximating the pulse by 10 hard pulses at a 100 μs interval. In the case of the transient-state experiment, the approximation was incorporated in the Bloch simulation in a straightforward manner. In case of the hybrid-state model, the RF-pulse was implemented as a linear ramp of *ϑ*. In case of the the steady-state sequence, the finite pulse correction prohibits the use of the standard steady-state equations. Therefore, we used the hybrid state framework and simulated the signal with a constant *ϑ* and used the signal after 1000 repetitions as the steady-state signal.

Mean and standard deviation were calculated. Due to the high input SNR in this non-imaging experiment, the standard deviation was multiplied a factor of 10 for easier depiction in Fig. 4. This set of experiments was repeated while manipulating one linear component of the shim coils in order to increase the width of the Larmor-frequency distribution.

### In vivo experiments.

An asymptomatic volunteer’s brain was imaged following written informed consent and according to a protocol approved by our institutional review board. A measurement was performed with the anti-periodic bHSFP experiment on a 3T Prisma scanner (Siemens, Erlangen, Germany). The manufacturer’s 20 channel head/neck coil was used for signal reception.

Spatial encoding was performed with a sagittally oriented 3D stack-of-stars trajectory, which starts at the outer *k*-space and acquires for one *T*_C_ data while incrementing the angle of the *k*-space spoke by twice the golden angle increment^54^. These large gaps were filled by repeating this procedure one time with the entire *k*-space trajectory rotated by the golden angle. Thereafter, the next 3D phase encoding step was performed in the exact same way, while adhering to the Nyquist–Shannon theorem along the slice direction. The acquired resolution of the maps is 1 mm × 1 mm × 2 mm at a FOV of 512 mm × 512 mm × 192 mm. The readout dwell time was set to 2.1 μs. We used a *T*_R_ = 4.5 ms and the readout was skipped in segments with a polar angle close to zero (gray areas in Supplementary Fig. 5), so that 601 spokes were acquired during one *T*_C_. The total scan time was ~12.24 min.

Along the fully sampled phase encoding direction, a Fourier transformation was performed and, thereafter, each slice was treated separately. Image reconstruction was performed with the low rank alternating direction method of multipliers (ADMM) approach proposed in ref.^55^, which includes parallel imaging^56–58^ and avoids undersampling errors typical for MRF^59,60^. The data consistency step of the ADMM algorithm was performed with 100 conjugate gradient steps. In order to prevent non-linear effects from impairing the noise assessment, only a single ADMM iteration was performed and no spatial regularization was applied.

The low rank approximation was calculated based on a coarse dictionary that covers the range between 100 ms and 6 s in steps of 10% and *T*_2_ between 10 ms and 3 s also in steps of 10%. The dictionary further discretized *ϕ* ∈ [0, *π*] into 15 bins and $B_{1}/B_{1}^{\text{nom.}}\in [0.7,1.2]$ into 40 bins. For consistency, we calculated a dictionary with Bloch simulations and used it to calculate a rank 6 approximation of the data with a singular value decomposition of the dictionary matrix^61^.

Represented in this low rank approximation, we fitted each voxel of the data with a non-linear least-square fit while accounting for the finite RF-pulse duration. We fixed *ϕ* and $B_{1}/B_{1}^{\text{nom.}}$ for each voxel to the values that resulted from separate scans^27^. The *ϕ* map was acquired with a double-echo SPGR experiment and the *B*_1_ map with a turboFLASH experiment, as described in ref.^41^.

## Supplementary Material

Supplemental Notes
