# Supplementary material for: Hybrid-state free precession in nuclear magnetic resonance
Source: Commun Phys. Author manuscript; Available in PMC 2019 Jul 19. (PMC6641569; doi:10.1038/s42005-019-0174-0)
Supplement: Supplemental Notes [file NIHMS1036806-supplement-Supplemental_Notes.pdf]

Supplementary Note 1. Relation of the Hybrid State to Literature

IR-bSSFP as a Special Case of IR-bHSFP

In the following, we will assume a constant ϑ, which converts a IR-bHSFP to a standard IR-bSSFP experiment[1] and allows us to solve the integrals in Eq. (7):

$$r(t) = \exp\left(-t/T_1^*\right) \cdot \left(r_0 - \frac{\cos\vartheta}{\frac{T_1}{T_2} \cdot \sin^2\vartheta + \cos^2\vartheta} \right) + \frac{\cos\vartheta}{\frac{T_1}{T_2} \cdot \sin^2\vartheta + \cos^2\vartheta} \tag{1}$$

with

$$\frac{1}{T_1^*} = \frac{\sin^2\vartheta}{T_2} + \frac{\cos^2\vartheta}{T_1}. \tag{2}$$

Transforming back to Cartesian coordinates with $S(t) = r(t) \cdot \sin\vartheta$, the signal is given by

$$S(t) = \exp\left(-t/T_1^*\right) \cdot \left(S_0 - S_{stst}\right) + S_{stst} \tag{3}$$

with the initial signal $S_0 = r_0 \cdot \sin\vartheta$ (with $r_0 = -1$ for an inversion recovery experiment). The steady state signal is given by

$$S_{stst} = \frac{\sin\alpha}{\left(\frac{T_1}{T_2} + 1\right) - \cos\alpha \left(\frac{T_1}{T_2} - 1\right)} \tag{4}$$

with the flip angle $\alpha = 2\vartheta$. The trigonometric identities $\sin(2\vartheta) = 2\sin\vartheta\cos\vartheta$ and $\cos(2\vartheta) = \cos^2\vartheta - \sin^2\vartheta$ were used for this transformation. Eqs. (2), (3) and (4) describe an exponential decay and are equivalent to Eqs. (2), (6) and (5) in Ref.[1]. Further, $1/T_1^*$ is equivalent to $R_{||}$ in Ref.[2] when assuming on-resonance ($\phi = \pi$).

Pseudo-Steady State Free Precession

Ref.[3] names three conditions under which the spin-echo nature of bSSFP experiments[4] is maintained when varying the flip angle. The first of these *pseudo-SSFP conditions* effectively limits $\Delta\alpha$, and the hybrid-state adiabaticity condition is the stricter limit. The other two pseudo-SSFP conditions define a specific T_R-pattern to ensure the correct timing for the spin echo formation. The strict limitation of $\Delta\alpha$ and $\Delta\phi$ in the hybrid-state framework allow to approximate these pseudo-SSFP conditions by a constant T_R, such that hybrid-state experiments are a sub-set of pseudo-SSFP experiments.

The stricter conditions of the hybrid state also result in a more benign magnetization response. While the hybrid state eliminates the perpendicular component, the pseudo-SSFP approach strives to control it. As a result, the spectral response of pseudo-SSFP experiments is smooth only in the neighborhood of the on-resonant spin isochromat[3]. With increasing off-resonance, this property starts to fail, which makes the signal at those frequencies sensitive to inhomogeneous broadening. In contrast, the hybrid-state maintains this property at all Larmor frequencies.

Supplementary Note 2. Robustness of the Hybrid State to Field Inhomogeneities

As pointed out in the main paper, the fully-transient state can be very sensitive small deviations in the B_0 field, which dictates the Larmor frequency, and the B_1 field, which dictates the Rabi frequency. In order to demonstrate this, we simulated the magnetization response to a train of uniformly distributed random flip angles in the range $\alpha \in [0, \pi]$ with $\phi = 0$. The simulations assume both fields to be perfectly homogeneous and we performed two sets of simulations where we assume B_0 to be perfectly calibrated and we vary the B_1 and vice versa. For such an experiment, a B_1-deviation of only 6% can cause T_1 to be overestimated by more than three orders of magnitude in the mean value with a tremendous standard deviation (Supplementary Fig. 1a). When we vary B_0, we observe that an off-resonance frequency of about 50Hz (which corresponds to approximately $\Delta\phi = 0.5\pi$) can cause both relaxation times to be over- or underestimated by several orders of magnitude and this bias is highly non-monotonous (Supplementary Fig. 1b,d). For such errors, a correction becomes extremely difficult, since unavoidable errors in the calibration scan result in severe and hard to predict errors in the estimated relaxation times.

Note that this random flip angle pattern is an extreme case that was deliberately chosen to highlight the danger of a careless experiment design in the transient state. In the cases we studied so far, we observed that the hybrid state fails gracefully in a sense that a small violation of the hybrid state condition (Eq. (4)) results in a slight increase of the sensitivity to magnetic field inhomogeneities, so that we can correct for deviations of B_0 and B_1. This leaves inhomogeneous broadening as the main source of biases in the absence of an adequate model and external calibration measurements (Fig. 3). Yet, from a theoretical point of view, we found no guarantee for graceful failure.

Supplementary Note 3. Numerical Optimizations

The Cramér-Rao Bound in Parameter Space

In the main article, the *rCRB* was analyzed only at the specific relaxation times used during the optimization. Supplementary Fig. 2 analyzes the experiments that were optimized for $T_1 = 781$ ms and $T_2 = 65$ ms in a larger parameter space, i.e. over a larger range of T_1 and T_2 values. The analyzed experiments were simultaneously optimized for T_1 and T_2. In this analysis, however, we examine $rCRB(T_1)$ and $rCRB(T_2)$ separately. Note that the relative Cramér-Rao bounds, as defined in Eqs. (39) and (40), take the correlation between the different parameters into account.

Supplementary Fig. 2 confirms the improved encoding power of the hybrid state in comparison to exponential decay curves and the steady state (Fig. 5 in the main article). Especially the $rCRB(T_2)$ is reduced when using the IR-bHSFP experiment instead of the exponential one, but also the $rCRB(T_1)$ is improved for long T_1 and short

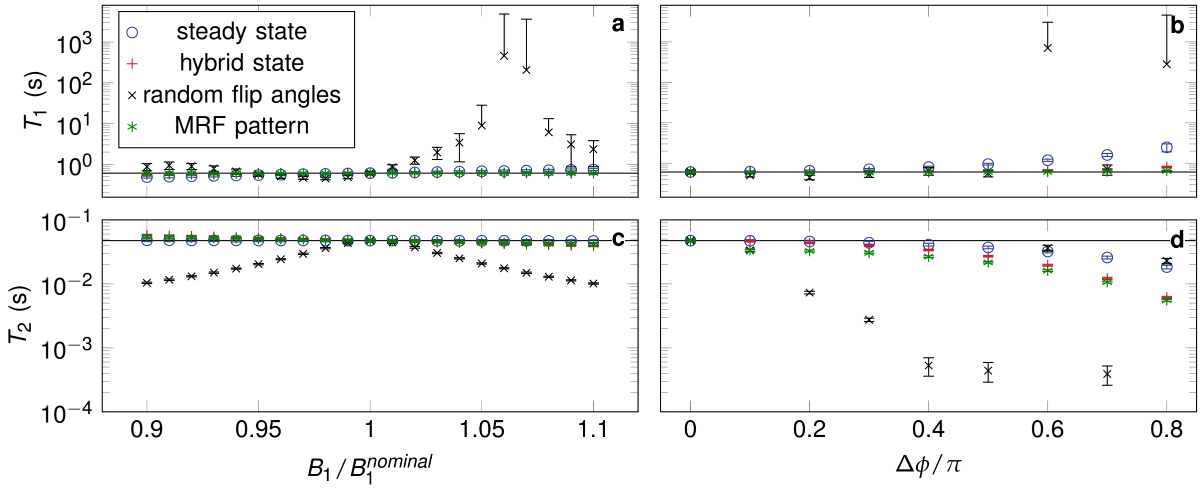

Supplementary Figure 1: Simulation-based accuracy and precision analysis of relaxation times measurements in different spin ensemble states. **a**,**c** In order to highlight potential sensitivity of the fully-transient state to B_1 inhomogeneities, we show relaxation times (mean and standard deviation) estimated from signal generated by a random flip angle pattern in comparison to the experiments described in the main article. **b**,**d** repeats a,c for B_0 inhomogeneities. The data were simulated under the assumption of homogeneous fields and we assume one field to be perfectly calibrated while varying the other. On can observe that the random flip angle pattern—as an extreme example of the transient state—can result in estimated relaxation times that deviate by several orders of magnitude from the ground truth (horizontal line), while the steady state, the hybrid state, and the MR-Fingerprinting pattern show a more benign response. The latter serves as a transient-state example that violates the hybrid-state condition (Eq. (4)) only slightly.

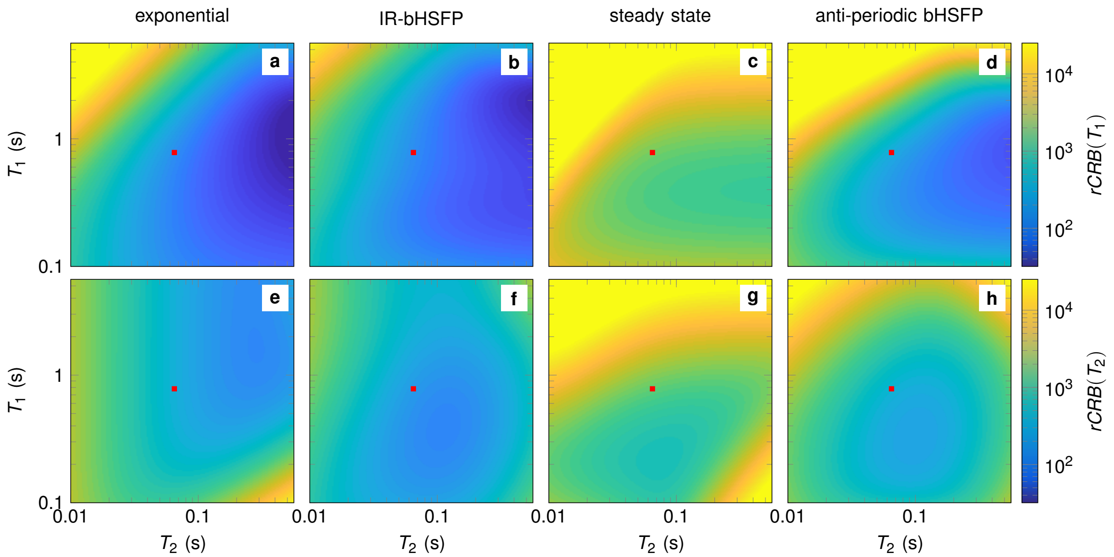

Supplementary Figure 2: Relative Cramér-Rao bounds in T_1-T_2-space. The performance of the optimized experiments is illustrated through plots of the relative Cramér-Rao bounds, which provide a lower bound for the noise in the retrieved relaxation times. All patterns were optimized for $T_1 = 781$ ms and $T_2 = 65$ ms, as indicated by the red square, and were tested for the entire parameter space in a sample MRF dictionary. The experiments have a $T_C = 3.8$s. Note the logarithmic scale in all three dimensions.

T_2 (top left corner in Supplementary Fig. 2b vs. a). A similar observation can be made when comparing the steady-state experiment to the anti-periodic hybrid-state one. In most of the areas of the T_1-T_2-space, the *rCRB* of the hybrid state is superior.

The Cramér-Rao Bound in the Presence of B_0- and B_1-inhomogeneities

Spatial variations of the main magnetic field (B_0) are an inevitable problem in NMR, especially in MRI. In the present framework, they are captured by ϕ and affect the spin dynamics as detailed in Eqs. (5)-(7). The radio-frequency field used for spin excitation (B_1) is also subject to variations. In general, B_1 inhomogeneities scale the flip angle of RF pulses linearly and cause variations of the polar angle (Eq. (5)) and consequently of r, as described by Eq. (7).

The experiments' encoding power for T_1 and T_2 is analyzed in Supplementary Fig. 3 for different B_0- and B_1-values under the assumption that the B_0 and B_1 variations are known from a separate measurement. Hardly any variation of the *rCRB* can be observed for any of the experiments within the analyzed range of parameters,

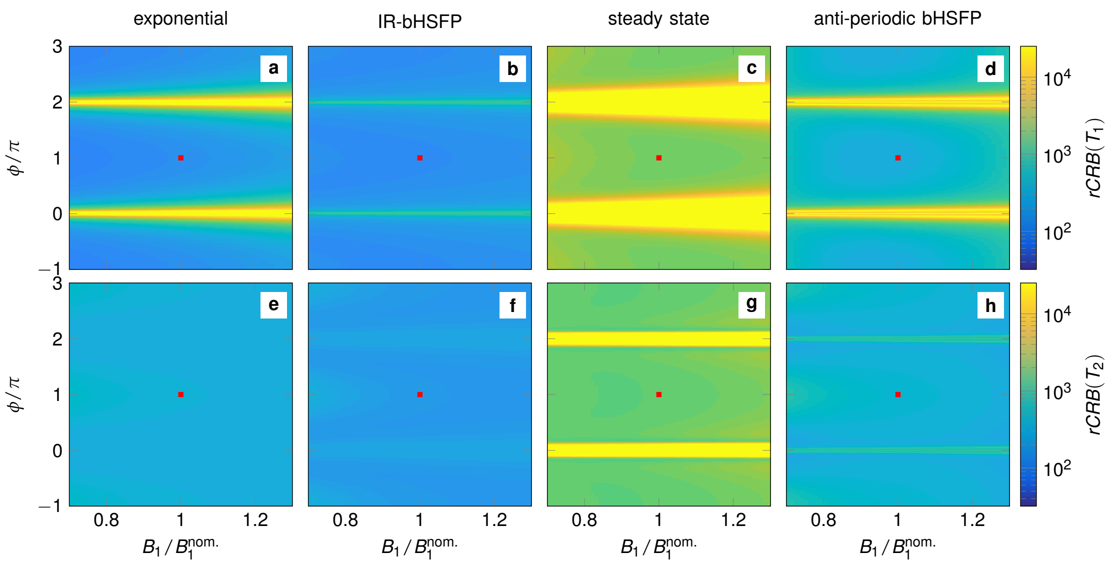

Supplementary Figure 3: Relative Cramér-Rao bounds in B_0-B_1-space. The performance of the optimized experiments is illustrated as a function of the main magnetic field, which is parameterized by the phase ϕ, and of the inhomogeneities of the magnetic field B_1, which is used for spin excitation. The relaxation times were fixed to $T_1 = 781$ ms and $T_2 = 65$ ms, which are the values used during the optimization. The experiments have a $T_C = 3.8$s. The red square indicates the nominal values.

apart from the vicinity of the stop-band ($|\sin\phi| \ll 1$). In the latter case, the magnetization lives close to the x-y-plane ($\vartheta \approx \pi/2$) for any $\sin^2\alpha \gg \delta$ (Eq. (5)), such that the magnetization is almost brought to naught (Eq. (7)). Intuitively, this leads a poor encoding of the parameters. In the case of the optimized steady-state experiment, the affected frequency band is larger compared to the hybrid-state experiments, in particular for T_1 (Supplementary Fig. 3c vs. d). B_1-inhomogeneities seem to have a minor impact on the T_1 encoding capabilities with the biggest degradation observed in the anti-periodic hybrid-state experiment. Note that Figs. 3d,h do reflect the imperfection of the inversion pulse (Eq. (38)). For T_2, the effect of the B_1 inhomogeneities is slightly larger and the worst $rCRB(T_2)$ in all experiments is located at small B_1 values and an improvement of the $rCRB$ compared to $B_1/B_1^{\mathrm{nom.}} = 1$ can be observed at high B_1-values, reflecting the limit $\vartheta \leq \pi/4$, which was used during the optimization process and is exceeded for $B_1/B_1^{\mathrm{nom.}} > 1$.

Spin Dynamics

For comparison, the original MRF[5] pattern is depicted in Supplementary Fig. 4a-c and represents a heuristic guess in the fully-transient state. Note that we depict here only the central isochromat and the dynamics of the other isochromats vary strongly. The original pseudo-SSFP pattern[3] is a heuristic guess that approximates the hybrid state (Supplementary Note 1), but inherits some fluctuations from the original MRF experiment (Supplementary Fig. 4d-f).

The inversion recovery (IR) bSSFP experiments is a special case of the hybrid state, which forces the magnetization onto a straight line that goes from the southern half of the unit-sphere to the steady-state ellipse (Supplementary Fig. 4g). The depicted experiment was optimized by a global search for the constant ϑ with the lowest relative Cramér-Rao bound.

Supplementary Fig. 4j-l show a hybrid-state experiment with periodic boundary conditions, which was optimized using the limit $0 \leq \vartheta \leq \pi/4$ for practical reasons. This effectively forces the magnetization to stay on the northern hemisphere, and the optimized trajectory splits into three segments: Starting from large z-values, the magnetization is excited to large ϑ-values, where the spin dynamics is dominated by T_2-relaxation. Thereafter, the magnetization follows a small loop close to the origin, which combines T_1- and T_2-encoding. In the last segment, the magnetization stays close to the z-axis, where the dynamics is dominated by T_1-relaxation, before the cycle starts over again. The periodic boundary condition enforces $r(0) = r(T_C)$. The optimizations also result in $\vartheta(0) \approx \vartheta(T_C)$, which is not enforced, but rather reflects the apparent optimality of smooth RF-pattern and ensures adherence to Eq. (4), similarly to the anti-periodic pattern (Fig. 6d,e).

The steady-state experiment depicted in Supplementary Fig. 4m-o was optimized with an unbound polar angle. However, the steady-state model limits the search to the steady-state ellipse. As demonstrated by Eq. (4), the steady-state signal of bSSFP experiments depends only on the ratio T_1/T_2. In order to disentangle T_1 and T_2, one commonly combines the bSSFP experiment with a gradient- and RF-spoiled gradient echo (SPGR) segment, whose steady-state magnetization does not depend on T_2[6]. The steady-state magnetization of the SPGR experiment lies on a different ellipse that depends on T_R and T_1 (inner ellipse in Supplementary Fig. 4m). Optimizations based on the steady-state model results in three discreet polar angles, one in the SPGR segment and two in

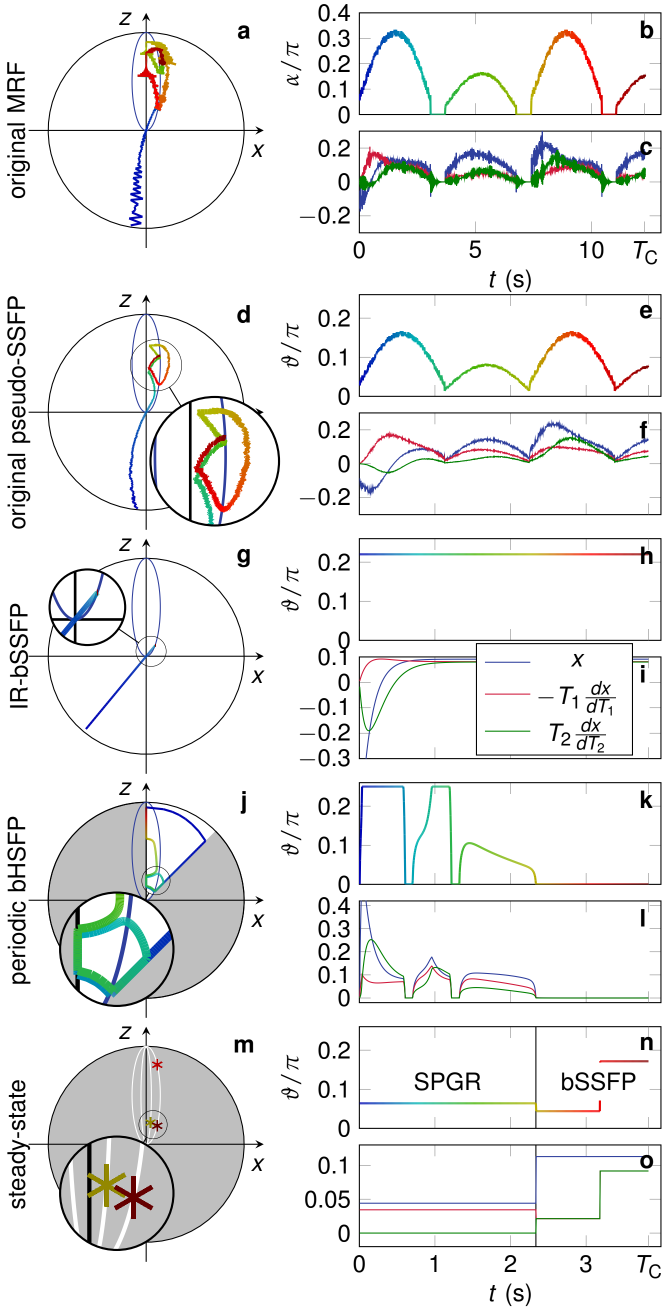

Supplementary Figure 4: Comparison of the spin dynamics for various experiments. **a** Spin ensemble trajectory of the magnetic resonance fingerprinting (MRF) experiment on Bloch the sphere. **b** Flip angle pattern of the MRF experiment, where the color scale provides a reference for the trajectories on the Bloch spheres. **c** The transversal magnetization and its normalized derivatives with respect to the relaxation times, which are the foundation of computing the relative Cramér-Rao bound. **d**-**o** Repeats a-c for various other experiments. The original MRF and pseudo steady-state free precession (pseudo-SSFP) experiments represent heuristic guesses, while the inversion recovery balanced steady-state free precession (bSSFP), the periodic balanced hybrid-state free precession (bHSFP), and the steady-state experiments were jointly optimized for T_1 and T_2. Note that the ϑ of the bSSFP segment in (j-l) were retrospectively sorted in increasing order.

the bSSFP segment (Supplementary Fig. 4n). The polar angles in the bSSFP segment have the same signal intensity (Supplementary Fig. 4o), which was already

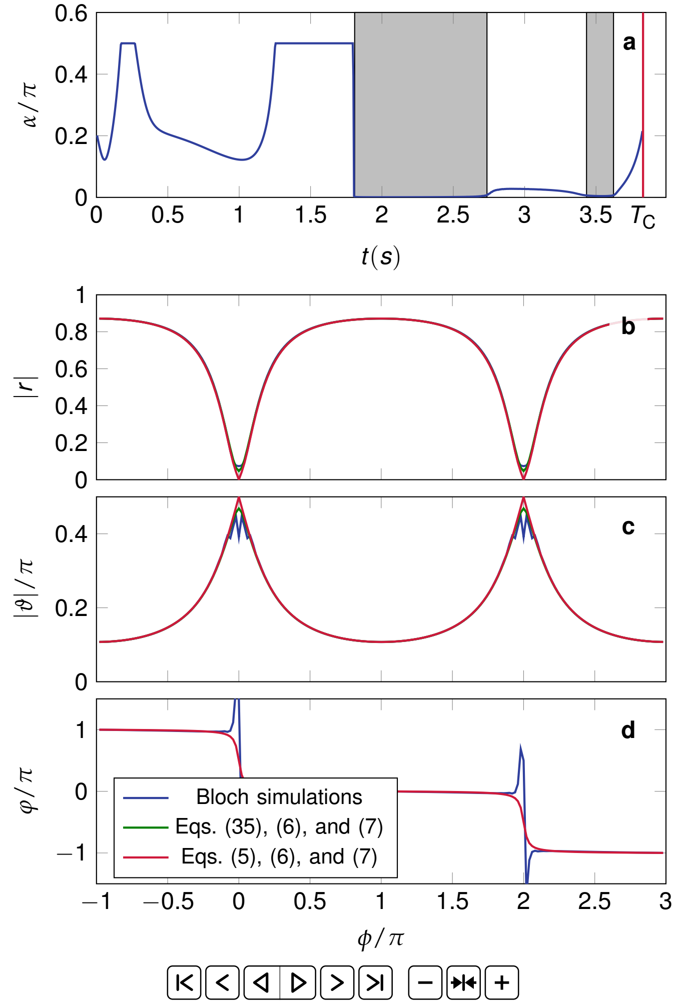

Supplementary Figure 5: Spectral response of a spin ensemble in hybrid state. The derived Eqs. (5)-(7) are verified against Bloch simulations (Eq. (1)) at the example of the optimized anti-periodic experiment. Additionally, Eq. (35) provides the exact solution of ϑ in steady state. In agreement with the approximate nature of the derivation, good accordance can be observed anywhere apart from the vicinity of the stop band, which is defined by $|\sin\phi| \ll 1$. The gray areas indicate time points that were not acquired in the in vivo scan since the polar angle is close to zero, which violates assumptions made in the derivation and results in negligible signal. This figure assumes $\phi_{T_E} = \pi/2$, which is commonly achieved by setting $T_E = T_R/2$ in experiments with balanced gradient moments. *In order to run the animation, please use a PDF-viewer capable of JavaScript, such as the Adobe Acrobat Reader.*

found to be optimal in literature[6]. Different from literature is, however, the single flip angle in the SPGR segment. In Ref.[6], the signal from two SPGR flip angles was utilized to quantify T_1 independent of the bSSFP segment. Accounting for the bSSFP segment in the T_1 quantification, the numerical optimization results in a single flip angle for all time points in the SPGR experiment, or in total in three different flip angles for disentangling three parameters (T_1, T_2 and proton density). Note that the T_2'-decay in the SPGR segment is neglected here.

Spectral Response

Eq. (5) provides a variety of combinations of ϕ and α that result in the desired ϑ-pattern. Throughout this

paper, we set $\phi = \pi$ for the central Larmor frequency, which requires $\alpha = 2\vartheta$ for all hybrid-state experiments and the bSSFP segment of the steady-state experiment, while $\alpha = \vartheta$ correctly describes the SPGR segment. Supplementary Fig. 5 verifies Eqs. (5)-(7) for anti-periodic boundary conditions by depicting the spectral response of the magnetization. Good accordance can be observed between the hybrid-state model and the Bloch simulations apart from the vicinity of the stop-band ($|\sin\phi| \ll 1$), which is in agreement with the approximations made in the derivation. In general, the depicted spectral response is smooth and the phase is almost constant within each pass band, which connects the absence of inhomogeneous broadening to the spin-echo-like behavior known for bSSFP experiments[3,4]. One can observe some fluctuations of ϑ and φ in the Bloch simulation around the time of the zero-crossing ($t \approx 0.3$s), i.e. when $r \approx 0$. Note that these errors are negligible in Cartesian coordinates due to the small absolute value of the magnetization.

One can further observe how the hybrid-state model breaks down in the gray segments in Supplementary Fig. 5. The rapid decrease in the flip angle at the beginning of the first gray segment violates the adiabaticity condition (Eq. (4)) so that we observe significant contributions of the orthogonal eigenstates. However, the expected hybrid-state signal is small because these segments fulfill $\vartheta \ll 1$. Consequently, we can simply discard the signal measured in these segments and by the end of the gray segment the population of the orthogonal eigenstates is sufficiently decayed. Note that the phase φ is not well defined for $\vartheta \approx 0$, causing strong fluctuations of the phase at small polar angles. The condition $\alpha^2 \ll \delta$ is not fulfilled in the gray segments in Supplementary Fig. 5, which was required for the eigenvector analysis[2]. Nevertheless, after the initial decay in the first gray segment, and throughout the second gray segment, we observe a residual error mostly in the vicinity of the stop band. The combination of a small α and a small ϕ results in a non-negligible factor ξ in Eq. (36), making Eq. (5) invalid. However, using the exact solution of the steady-state ϑ (Eq. (35)) substantially mitigates these errors (green graphs in Supplementary Fig. 5).

Supplementary Note 4. In Vivo Experiments

Supplementary Fig. 6 supplements Fig. 7 and shows all slices of the 3D dataset, acquired with the anti-periodic bHSFP experiment.

Additionally, we imaged an asymptomatic volunteer's knee following written informed consent and according to a protocol approved by our institutional review board (Supplementary Fig. 7). A measurement was performed with the anti-periodic bHSFP experiment on a 3T Prisma scanner (Siemens, Erlangen, Germany). A commercial transmit-receive knee coil with 15 receive elements was used. The images were acquired with a spatial resolution of 1 mm $\times$ 1 mm $\times$ 1 mm and an RF-pulse duration

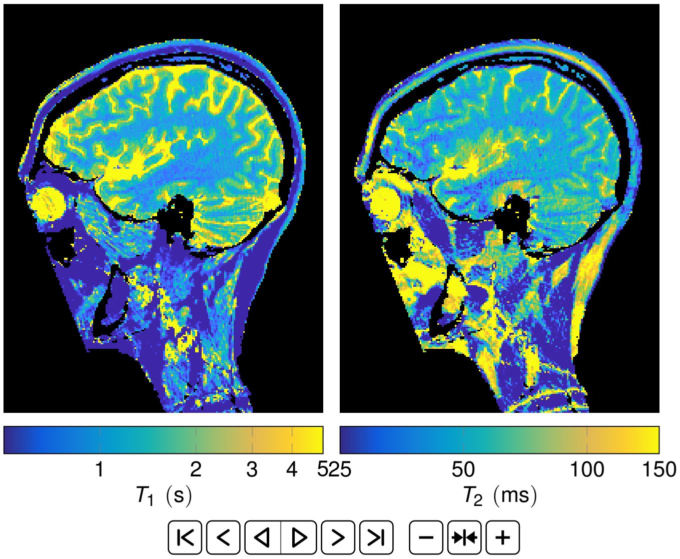

Supplementary Figure 6: Quantitative spin relaxation times measured in hybrid state. All slices of the in vivo 3D data set are depicted. The data were acquired with the anti-periodic HSFP experiment and were fitted with the hybrid-state model (Eqs. (5) and (7)). The parameter maps have a resolution of 1 mm $\times$ 1 mm $\times$ 2 mm. Note the logarithmic scale of the T_1 and T_2 color map. The face was cut off in order to protect the volunteer's identity. *In order to run the animation, please use a PDF-viewer capable of JavaScript, such as the Adobe Acrobat Reader.*

of 350µs, which was approximated by 7 hard pulses separated by 50µs in the fitting routine. Otherwise, the same acquisition and reconstruction parameters were used as for the brain images (Fig. 7 and Supplementary Fig. 6).

The parameter maps in the knee are qualitatively good and the relaxation times in the articular cartilage are similar to previously reported values[7].

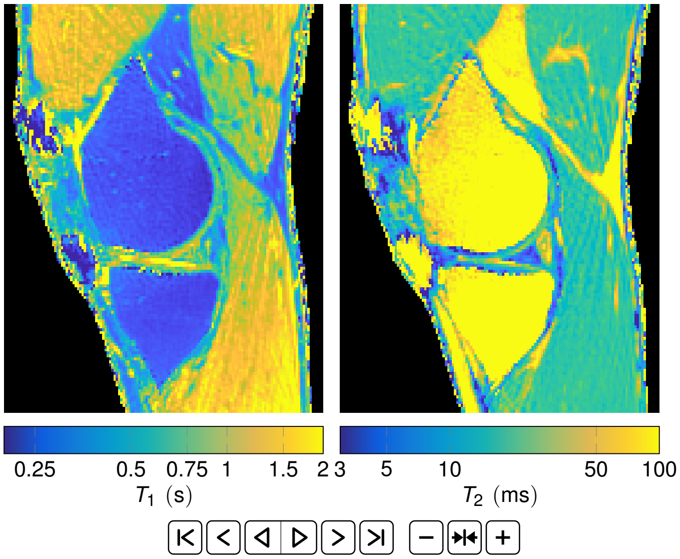

Supplementary Figure 7: Quantitative spin relaxation times measured in hybrid state. The sagittal slices of the in vivo 3D data set of a human knee were acquired with the anti-periodic HSFP experiment depicted in Fig. 6e and fitted with the hybrid-state model (Eqs. (5) and (7)). The parameter maps have an isotropic resolution of 1mm. Note the logarithmic scale of the T_1 and T_2 color map. *In order to run the animation, please use a PDF-viewer capable of JavaScript, such as the Adobe Acrobat Reader.*

Supplementary References

[1] Schmitt, P. *et al.* Inversion recovery TrueFISP: quantification of T1, T2, and spin density. *Magnetic Resonance in Medicine* **51**, 661–667 (2004). URL http://www.ncbi.nlm.nih.gov/pubmed/15065237.

[2] Ganter, C. Off-resonance effects in the transient response of SSFP sequences. *Magnetic Resonance in Medicine* **52**, 368–375 (2004). URL http://doi.wiley.com/10.1002/mrm.20173.

[3] Assländer, J., Glaser, S. J. & Hennig, J. Pseudo Steady-State Free Precession for MR-Fingerprinting. *Magnetic Resonance in Medicine* **77**, 1151–1161 (2017). URL http://doi.wiley.com/10.1002/mrm.26202.

[4] Scheffler, K. & Hennig, J. Is TrueFISP a gradient-echo or a spin-echo sequence? *Magnetic Resonance in Medicine* **49**, 395–397 (2003). URL http://doi.wiley.com/10.1002/mrm.10351.

[5] Ma, D. *et al.* Magnetic resonance fingerprinting. *Nature* **495**, 187–192 (2013). URL http://www.nature.com/nature/journal/v495/n7440/full/nature11971.html.

[6] Deoni, S. C. L., Rutt, B. K. & Peters, T. M. Rapid combined T1 and T2 mapping using gradient recalled acquisition in the steady state. *Magnetic Resonance in Medicine* **49**, 515–526 (2003).

[7] Cloos, M. A. *et al.* Rapid Radial T 1 and T 2 Mapping of the Hip Articular Cartilage With Magnetic Resonance Fingerprinting. *Journal of Magnetic Resonance Imaging* 1–6 (2018). URL http://doi.wiley.com/10.1002/jmri.26615.
